# Insight into the regulatory networks underlying the high lipid perennial ryegrass growth under different irradiances

**DOI:** 10.1371/journal.pone.0275503

**Published:** 2022-10-13

**Authors:** Somrutai Winichayakul, Richard Macknight, Liam Le Lievre, Zac Beechey-Gradwell, Robyn Lee, Luke Cooney, Hong Xue, Tracey Crowther, Philip Anderson, Kim Richardson, Xiuying Zou, Dorothy Maher, Gregory Bryan, Nick Roberts

**Affiliations:** 1 AgResearch Ltd., Palmerston North, New Zealand; 2 Department of Biochemistry, University of Otago, Dunedin, New Zealand; Kyung Hee Univeristy, REPUBLIC OF KOREA

## Abstract

Under favourable conditions, perennial ryegrass (*Lolium perenne*) engineered to accumulated high lipid (HL) carbon sink in their leaves was previously shown to also enhance photosynthesis and growth. The greater aboveground biomass was found to be diminished in a dense canopy compared to spaced pots. Besides, the underlying genetic regulatory network linking between leaf lipid sinks and these physiological changes remains unknown. In this study, we demonstrated that the growth advantage was not displayed in HL Lolium grown in spaced pots under low lights. Under standard lights, analysis of differentiating transcripts in HL Lolium reveals that the plants had elevated transcripts involved in lipid metabolism, light capturing, photosynthesis, and sugar signalling while reduced expression of genes participating in sugar biosynthesis and transportation. The plants also had altered several transcripts involved in mitochondrial oxidative respiration and redox potential. Many of the above upregulated or downregulated transcript levels were found to be complemented by growing the plants under low light. Overall, this study emphasizes the importance of carbon and energy homeostatic regulatory mechanisms to overall productivity of the HL Lolium through photosynthesis, most of which are significantly impacted by low irradiances.

## Introduction

The climate crisis and increasing world population mean dramatic increases in the production of food, forage and renewable fuels is needed using the existing agricultural land. To address this, we and others have been exploring biotechnological approaches to increase the levels of energy-rich triacylglycerol (TAG) content in plant non-seed tissues [1,2]. Increased TAG synthesis in vegetative tissues can be achieved by promoting the carbon flux into fatty acid (FA) and TAG synthesis through the manipulation of enzymes and/or transcriptional regulators whilst suppressing FA catabolism, including FA utilization and TAG turnover [3–5]. We previously reported the development of transgenic lines of high-lipid (HL) perennial ryegrass (*Lolium perenne*) and Arabidopsis, that co-express diacylglycerol O-acyltransferase (DGAT, the enzyme for TAG biosynthesis) and cysteine-oleosin (Cys-OLE, a modified oleosin designed to protect lipid droplets from proteolysis). These plants had up to twice the FA content in mature leaves (from ~ 3.5% to ~ 7% dry weight (DW)) as well as increased plant CO_2_ assimilation rate and biomass [6–12]. To explain this secondary growth benefit, Beechey-Gradwell et al. [10] speculated the trade-off between lipids and sugars in HL leaves might alter the leaf sugar homeostasis, which in turn mitigates negative feedback on photosynthesis [13].

During the day, plants fix inorganic CO_2_ by carboxylating ribulose-bisphosphate, producing phosphorylated intermediates which is subsequently partitioning into several central metabolic pathways, including the generation of carbohydrates, proteins and lipids [1,14]. Throughout the day/night cycle, plants breakdown assimilated carbon through respiration for growth and maintenance [15]. The intracellular availability of sugars is well recognized for its roles in communication between sugar sensing and redox chemistry [16–18]. Therefore, diverting carbon allocation from sugars to lipids, in HL plants, likely alters other metabolisms in the plant cells. Paul and Eastmond [13] suggested that lipid carbon sink sequesters carbon away from sugar-sensing mechanisms and proposed if the elements of the trehalose 6-phosphate (T6P)/sucrose non-fermented related kinase 1 (SnRK1) signalling complexes may be altered in HL leaves. Expression of genes involved in photosynthesis were shown to be activated by SnRK1 overexpression although there is limited evidence for a direct link of this signalling pathway [19,20]. Antagonistically to SnRK1 activity, T6P elevation by overexpressing T6P synthase 1 (TPS1), an enzyme involved in T6P synthesis, can positively impacted photosynthesis [21]. However, rather than have a direct sink effect on photosynthesis, T6P changes the distribution of photoassimilates in phloem cells through SWEET genes, triggering sugar demand within the photosynthesis cells [22].

Likewise, promoting *de novo* FA biosynthesis places higher demands for both energy and reducing power in the form of NADPH and NADH than those required for complex carbohydrate synthesis [23]. This, therefore, can also disturb a balance between the generation and consumption of reductants through photochemical reactions within different plant organelles. Without CO_2_ recycling by Rubisco, carbon conversion efficiency for *de novo* lipid biosynthesis is much lower albeit the energy density of lipids is more than twice of carbohydrates [8,10,24]. How do lipid carbon sink plants improve their solar energy conversion into plant biomass and balance their energy homeostasis? The metabolizing cells of growing leaves actively trap excess incoming light energy productively for assimilatory processes and manipulate the non-photochemical quenching to maximize the primary productivity [25]. Components of these major metabolic pathways including the Calvin-Benson cycle, the malate valves, the respiratory alternative oxidases, and the oxidative pentose phosphate pathways, as well as glycolysis are tightly linked to sensing of energy fluxes and imbalance responses [26]. This study provides an insight into the impact of sink manipulation on carbon capture and partitioning at the genetic level of regulatory mechanisms participated in carbon and energy homeostasis.

The interconnection between sugar and lipid metabolism appears to be in a delicate metabolic equilibrium that can also be orchestrated in response to changing environments [27,28]. For the commercial breeding purposes, it is crucial to understand how the HL Lolium performs and acclimates to the prevailing environment in the field, particularly those experienced under sward conditions [29]. In our two field trials in Missouri USA, the enhanced lipid trait was confirmed in HL mini-sward canopies grown in the field, but the growth benefit previously observed in the glasshouse, was absent [30]. Of the environmental factors, we speculated that low light (such as in the sward canopy) is a factor that has a substantial effect on photosynthesis over the day. Low light intensity directly influences photosynthesis and photomorphogenesis, which in turn impacts the agronomic traits of the plants. Low irradiance causes constraints of photosynthesis by lowering levels of PSII, Rubisco, electron transport rate, and CO_2_ assimilation [31]. The purpose of this study was to examine if low light is a key constraint for the growth performance of HL Lolium when growth temperature was optimized, and water availability was not limited [32]. In addition, the transcript profiling studies we present here provide a snapshot of the complex regulatory gene network that operates during lipid accumulation in the photosynthesis tissues, some of which were affected by light intensity.

## Materials and methods

### Experiments, plant establishment and growth

The expression cassette for all HL genotypes in this study contained the DGAT1 and Cys-OLE transgenes under the control of green-tissue specific *Rubisco small unit* and *chlorophyll a/b binding protein* promoters, respectively. The construct was designed and transformed as described in [12]. All transgenic genotypes used in this study differed genetically from the non-transformant (NT) only by the presence of the DGAT1/Cys-OLE construct. The transgenic genotypes were independent and differed by the transgene loci and copy numbers within the loci [10]. All experiments were conducted in controlled environment rooms (22/15 °C day/night temperature, 65–70% humidity, 12 h days) with a standard 600–1000 μmol m^-2^ s^-1^ PAR (SolarSystem 550, LED Grow Lights, NZ), unless otherwise stated. The NT and HL ramets consisting of 5 tillers, each were propagated into 1.3 L potting mix soil (Daltons, NZ) to generate multiple isogenic clones. To synchronize the plant growth, the propagation step was repeated three times with a 3-4-week gap between each step. Pots were rotated along the bench every 2–3 d to provide uniform exposure to the growth conditions.

For the main experiment described in this study, three T1 hemizygous HL transgenic genotypes ’HL30’, ’HL34’, and ’HL42’ and their respective NT plant (cultivar ’Impact’) were used in a preliminary regrowth trial for a detailed physiological comparison at standard and low (150–250 μmol m^-2^ s^-1^) PAR. In all experiments, clonal plantlets consisting of 5 tillers were used as biological replicates. These were generated by splitting up synchronized plants then cutting the tillers and attached roots to 10 cm of combined root and shoot length. These plantlets were transplanted into 1.3 L of washed coarse sand (Daltons, NZ), which was then completely flushed with a complete nutrient solution containing 2 mM of N as KNO_3_ [33]. The plantlets were then grown for three weeks in a controlled environment room with a 300–400 μmol m^-2^ s^-1^ PAR and 50 mL of nutrient solution, three times per week. At the end of this ’establishment period’, all plantlets were defoliated to 5–6 cm above the sand surface. Of the 40 plantlets generated from each parent genotype, the five smallest and five largest were discarded. Of the remaining 30 plantlets, a subset of 10 defoliated plantlets were destructively sampled and oven-dried at 65 °C for approximately 3–4 d. Total DW was determined as a DW_establish_, enabling the later calculation of relative growth rate (RGR). The remaining 20 clones were divided into two groups of 10 and randomly allocated to standard or low light treatment. These plantlets were regrown for an additional three weeks during which time they were provided with 50 mL of nutrient solution containing 4 mM NH_4_NO_3_ (8 mM total N), three times per week and every day during the final week of regrowth. One week before the final harvest, leaf-level photosynthetic parameters were measured.

Additional results included in this report are supplementary to support our further discussion on the differentiate transcripts of the HL Lolium in plant respiration. These experiments were previously conducted in T1 hemizygous ’Impact’ HL genotypes ’DGAT1+CO4’ and ’DGAT1+CO5’ [10,12]. Wax composition and photosynthesis-irradiance (P-I) curve and dark respiration in the light were measured from the plantlets grown in a potting mix soil (Daltons, NZ) in a controlled environment room with standard light through the 3-week establishment period and regrown for an additional three weeks after defoliated.

### Harvest, leaf water content and relative growth rate

Unless otherwise stated, plants were generally harvested at between 1–2 pm (5–6 h after starting of the day light) when the levels of leaf lipids (highest at the end of the night) and sugars (highest at the end of the day) are concomitantly at their average (S1 Fig and S1 Protocol). At final harvest, plants were destructively sampled and divided into ’leaf’ (leaf blade and leaf sheath from 6 cm above the sand surface), ’sheath’ (leaf sheaths on a tiller or pseudostem, 6 cm from the sand surface), and roots (cleaned). Small aliquots of leaf were subsampled, weighted for fresh weight (FW_1_), and oven-dried for dry weight (DW_1_). The remaining leaf material, sheath and roots were freeze-dried for 3–4 d for DW_final_. Leaf water content was calculated using a classical assessment based on the weight change between fresh and dried leaves as the following:

$$\text{Leaf}\;\text{water}\;\left(\%\right)=\left({\text{FW}}_{1}-{\text{DW}}_{1}\right)/{\text{FW}}_{1}*100$$

In this study, RGR is preferred over DW to eliminate ambiguous differences of DW data resulting from propagation. RGR was calculated from the average of DW_establish_ and individual DW_final_ as the following:

$$\text{Leaf}\;\text{RGR}=(\text{ln}\;\left[{\text{DW}}_{\text{final}}+{\text{DW}}_{1}\right]–\text{ln}\;\left[{\text{DW}}_{\text{establish}}\right)/\left({\text{t}}_{\text{final}}-{\text{t}}_{\text{establish}}\right)$$

where t is time between established and final harvests.


$$\text{Sheath}\;\text{or}\;\text{root}\;\text{RGR}=\left(\text{ln}\;\left[{\text{DW}}_{\text{final}}\right]–\text{ln}\left[{\text{DW}}_{\text{establish}}\right]\right)/\left({\text{t}}_{\text{final}}-{\text{t}}_{\text{establish}}\right)$$

### Lipid and sugar analysis

The lyophilized ground plant materials were analyzed for FA and sugars. FA were extracted from approximately 10 mg DW and methylated according to [34]. FA were verified and analyzed by gas chromatography-flame ionization detector (Shimadzu QP2010, Zebron^™^ ZB-FAME column (7FD-G033-05)) using Supelco^®^ 37 component FAME standard mix (Merck, CRM47885). The internal pentadecyclic acid (15:0, added prior to methylation) and methylated margaric acid (17:0) standards were used for quantification. Total FA concentration presented as percentage of plant dry weight are the sum of palmitic acid (16:0), palmitoleic acid (16:1), stearic acid (18:0), linoleic acid (18:1), linolenic acid (18:3), arachidic acid (20:0), gondoic acid (20:1), behenic acid (22:0), and erucic acid (22:1).

For water-soluble carbohydrate (WSC), approximately a 25 mg sample was extracted in 80% ethanol (v/v) and H_2_O for low molecular weight (LMW) and high molecular weight (HMW) -sugars, respectively [11]. The colourimetric reaction was developed using anthrone assay described in [35] and measured at 620 nm. Sugars were quantified using a dilution series of sucrose and inulin standards.

### SDS-PAGE analysis and immunoblot analysis

Protein samples were prepared by vigorously mixing 10 mg of lyophilized ground material with 150 μL of sterile H_2_O, 200 μL of 2X loading buffer (1:2 diluted 4X lithium dodecyl sulfate sample buffer [Thermo Fisher NP0007], 8 M urea, 4% [v/v] Triton X-100, and 5% [v/v] β-mercaptoethanol), and 40 μL of 10X reducing agent (Thermo Fisher NP0004). Equal volumes of soluble leaf protein were separated by SDS-PAGE on 4–15% gradient Mini-PROTEAN^®^ TGX stain-free^™^ precast gels; Bio-Rad system) and transferred to PVDF membranes (Bio-Rad Trans-blot Turbo system). Immunoblotting was performed as described in [8]. PVDF membranes were incubated with antibodies using the following and dilutions and manufacturers: 1:2500 anti-rabbit Cys-OLE (GenScript^®^); 1:1000 anti-malate dehydrogenase 2 (mitochondrial, AS15 3064, Agrisera); 1:1000 anti-cytochrome c oxidase (mitochondrial inner membrane, AS04 053A, Agrisera); 1:1000 anti-ascorbate oxidase (AS09 384, Agrisera); 1:2000 anti-L-ascorbate peroxidase (AS08 368, Agrisera); anti-trehalose-6-phosphate synthase 1 (AS12 2635, Agrisera); 1:2000 anti-SNF1-related protein kinase subunit γ (AS09 613, Agrisera); 1:5000 anti-rabbit IgG-HRP (Sigma, A6154). Protein-antibody complexes were developed using the Western Bright ECL spray (K12049-D50, Advansta, CA) and visualized by the ChemiDoc^™^ MP Imaging system (Bio-Rad). Volume intensity of protein bands was quantified using Image Lab^™^ software for PC version 5.2.1 (Bio-Rad).

### Chlorophyll concentration

Chlorophyll (Chl) was extracted from approximately 15 mg of lyophilized ground leaf materials with 3 mL of 95% [v/v] ethanol. Extraction tubes (sealed with Teflon lined screw cap) were kept at room temperature in the dark for 4 h with interval thoroughly mixing. The extracts were ¼ diluted before colorimetric measurement. The chlorophyll concentration in extracts was measured spectrophotometrically (VersaMax^™^ microplate reader) at the wavelengths of 648 nm and 664 nm and calculated for Chl_a_ and Chl_b_ contents, as described in [36]. Microplate absorbance from 200 μL of an ethanol extract was adjusted to 1-cm pathlength spectrophotometer with modification of 1/0.51 co-factors according to a report in [37].


$${\text{Chl}}_{\text{a}}\left(\mu \text{g}/\text{mL}\right)=\left(13.36\;{\text{A}}_{664}–5.19\;{\text{A}}_{648}\right)/0.51$$

$${\text{Chl}}_{\text{b}}\left(\mu \text{g}/\text{mL}\right)=\left(27.43\;{\text{A}}_{648}–8.12\;{\text{A}}_{664}\right)/0.51$$

$$\text{Chl}\left(\text{mmol}\;{\text{kg}}^{-1}\text{DW}\right)=12*\left[\left({\text{Chl}}_{\text{a}}/893.49\right)+\left({\text{Chl}}_{\text{b}}/907.47\right)\right]/\text{DW}$$

### Photosynthetic gas exchange

Three tillers were selected per plant, and on the youngest fully expanded leaves, net photosynthesis per unit leaf area, transpiration, stomatal conductance, electron transport rate and quantum efficiency of PSII was analyzed using a Licor 6800 infrared gas exchange system (Licor Biosciences Ltd., Nebraska, USA). Leaves were acclimated under growing conditions: 200 and 700 μmol photons m⁻^2^ s⁻¹ red/blue light for low and standard light treatments respectively, 400 ppm CO_2_, 70% relative humidity and 20 °C for 15 min before data-logging. The three leaves were then abscised, photographed, oven-dried and weighed. Leaf area was calculated using GIMP 2.8.22 (GNU Image Manipulation Program, http://www.gimp.org).

### P-I curves and dark respiration in the light

Three leaves were selected to measure the photosynthetic rate at different irradiance of 1.5, 50, 100, 200, 300, 500, 800, 1000, 1250, and 1500 μmol photons m^-2^ s^-1^. Dark respiration in the light (Rd) was measured separately via the Laisk method [38], determined from the intersection of A-Ci curves completed at 300, 100, 60 and 25 μmol photons m^-2^ s^-1^. For each plant, three leaves were acclimated in the leaf chamber under the following conditions: 300 μmol photons m⁻^2^ s⁻¹ red/blue light, 200 ppm CO_2_, 70% RH and 20 °C for 20 minutes. A-Ci curves at each irradiance were then performed using the following CO_2_ concentrations; 200, 170, 140, 110, 80, 50 ppm with three minutes of acclimation between each measurement.

### RNA isolation and sequencing

Approximately 50 mg of lyophilized ground leaf materials (three-weeks regrowth after defoliated) was used for RNA extraction with the Sigma-Aldrich Spectrum Plant Total RNA Kit, according to the manufacturer’s instructions. Total RNA (2–3 μg) was dried down and preserved in Sigma-Aldrich RNAstable^®^ before being couriered to BGI Tech Solutions (Hongkong) Co., Ltd. BGI Tech prepared and processed libraries for 20M PE100 reads using their DNBseq platform. The number of reads generated per sample was 49,052,411 ± 461,742. Adapters and low-quality reads were trimmed with TrimGalore, v0.6, using the following parameters: -paired, -retain unpaired, -phred33, -length 36 -q 5, -stringency 1 -e 0.1.

### Transcriptome assembly, expression analysis and annotation

The provided reads were assembled using Trinity v2.7.0 with a minimum contig length of 300 bp. To reduce sequence redundancy, assemblies were processed with CD-HIT-EST [39] with a threshold of 98% identity to remove nearly identical contigs. Further redundancy filtering was performed using the EvidentialGene tr2aacds script (Evidence directed gene construction for eukaryotes, https://sourceforge.net/projects/ evidentialgene/) which analyses transcripts for coding potential and generates three sets of transcripts; "okay-main" (transcripts), "okay-alt" (alternative transcripts), and "drops" (redundant or poorly informative transcripts). The "okay-main" transcript assembly was subsequently used for all downstream analyses. To check that the “okay-main” assembly retained a high level of completeness, the eukaryotic Benchmarking set of Universal Single-Copy Orthologs (BUSCO) analysis was performed using the ’transcriptome’ setting [40].

Transcript abundance and statistics were derived using BWA and Samtools. Differential expression levels were determined in R using DESeq and Biostrings. Differentially expressed genes (DEGs) were firstly selected for those with padj < 0.05. Local BLAST searches of the *Oryza sativa* IRGSP-1.0 and the *Brachypodium distachyon* databases provided best-hit identification of the differentially expressed contigs. Metabolic gene classes were taken as assigned by the KEGG database [41] and UniProt Knowledgebase. Annotation for Gene Ontology (GO) terms was done on final assembly using Blast2GO [42], and GO enrichment analysis was done using agriGO [43]. MapMan functional annotation and classification was generated from the final assembly using the online tool Mercator4 v4.0 [44]. Genes with adjusted p < 0.01 were presented using MapMan4.

### Quantitative real-time PCR

Total RNA was isolated from freeze-dried plant material using SpectrumTM Plant Total RNA (Sigma-Aldrich) according to the manufacturer’s protocol. RNase free DNase I (Roche) enzyme was used to remove the genomic DNA contamination in RNA samples. DNase treated RNA was subsequently purified using RNeasy Mini Spin Kit (Qiagen) to remove enzymes, salts and degraded DNA fragments. The absence of genomic DNA was confirmed by qPCR prior to reverse transcription. RNA quality and integrity was checked by 1% agarose gel electrophoresis. The concentration of RNA was measured by Qubit (Life technology). Two μg of RNA was reverse transcribed into cDNA using the SuperScriptTM IV VILO^™^ Master Mix (Invitrogen) following the manufacturer’s instructions. Primers for real-time PCR analysis of selected genes in *L*. *perenne* were designed using Primer3 (v.0.4.0) for a product length ranging between 130 and 170 bp. The sequences for these primers are listed in S1 Table. Ryegrass *transcription elongation factor 1* (*Lp-TEF1*) and *eukaryotic initiation factor-4α* (*Lp-eIF-4α*) genes were used as reference genes for the normalization of real-time data [45]. The PCR mixture contained 6 μl first strand cDNA (50 times diluted), Light Cycler ^®^480 SYBR Green I Master Mix (Roche Diagnostics NZ Ltd.), and 2 μM of each gene-specific primer in a final volume of 15 μl. Negative template controls were also performed for each of the primer pair. The real-time PCRs were performed employing Light Cycler ^®^480 real-time PCR machine (Roche Diagnostics NZ Ltd.). All the PCRs were performed under the following conditions: 5 min at 95°C, and 45 cycles of 10 s at 95 °C, 10 s at 60 °C, and 10 s at 72 °C. Melting curve analysis with a temperature gradient of 0.1 °C/s from 60 °C to 97 °C was performed to confirm that only the specific products were amplified. The fluorescence signal was measured at the end of each extension step at 72 °C. Three biological replicates were analyzed for each sample. The relative expression ratio was calculated using the advanced relative quantification program of Roche Light Cycler^®^480 software.

### Scanning electron microscopy of leaf surfaces

The epicuticular wax crystal of the adaxial leaf surfaces of ryegrass was characterized using scanning electron microscopy (SEM). At 3-week regrowth of plants following defoliation, 1 cm sections of fully mature leaf blade (tiller stage 4, leaf 2 position) [46] were sliced from the 3 cm measured from leaf collar region. Fresh leaves were attached onto a sticky carbon tab and air dried for 48 h. Samples were processed for SEM analysis at the Robinson Research Institute, Victoria University, Wellington, NZ.

### Analysis of cuticular wax composition by GC-MS

Cuticular waxes were extracted from approximately 0.5 g of fresh mature ryegrass leaves. The leaves were cut into 3.5 cm lengths and placed into glass test tubes containing 50 μg of internal standard heptadecane (C17 alkane, Supelco 51578). Six mL of heptane/toluene (1:1) was added into each tube and mixed well for 30 sec. The extraction solution was taken into a new glass test tube and evaporated under nitrogen gas at 37°C, followed by the addition of 100 μL of N,O‐bis(trimethylsilyl)-trifluoroacetamide with trimethylchlorosilane (Sigma, 15238) and 100 μL of pyridine (Sigma, 270970). The wax mixtures were heated at 90 °C for 30 min to convert to trimethylsilyl derivatives (silylation). The mixture was evaporated once more under nitrogen gas at 40 °C. The dried wax mixture was dissolved in 80 μL of heptane/toluene (1:1) and warmed to 37 °C before the wax aliquot was taken into a sample vial with glass insert. One μL of analyte was injected into the gas chromatograph mass spectrometry (GCMS-QP2010, Shimazu, Tokyo, Japan) for qualitative and quantitative analysis. The analysis conditions are as follows: column 60 m ZB-5, 0.32 mm i.d., df = 0.25 μm (7KM-G002-11, Zebron^™^), column temperature at 135 °C and held for 1.5 min, a rise of 15 °C min-1 to 220 °C and held for 4.5 min, an increase of 3°C min^–1^ to 290°C, held for 10 min, an increase of 2°C min^–1^ to 300°C, and held for 15 min. Injection temperature at 220 °C, split mode at split ratio 30.0, flow control mode: pressure at 150 kPa, total gas flow 77.6 mL/min, purge flow 3.0 mL/min.

### Statistical analysis

Experiment data were analyzed by Student t-test and one-way or two-way ANOVA using the RStudio version 3.6.0 with a model that included the fixed effect of ryegrass genotypes (HL and NT or null plants) and light intensities (standard and low lights). In some cases, a log-transformation was applied to the responses for matching the normality assumption of ANOVA. A multiple comparison of treatments such as Bartlett’s test (homogeneity of variances) and Shapiro-Wilk normality test from ANOVA was used to highlight significance among treatment means. P values were adjusted by the BH method [47] to control the false discovering rate. Means, SE and LSD are reported, and fixed effects declared significant at *p < 0.05, **p < 0.01, or ***p < 0.001).

## Results

### The influence of light intensity on FA and WSC levels in leaf and sheath

Table 1 shows FA levels for leaves and sheaths harvested at three weeks of regrowth. FA levels accumulated to a higher content in the leaves than in the sheath in all genotypes and under both light treatments. This result is not surprising given that only the outer layer of the sheath is fully photosynthetic. Compared to NT, all three HL lines had significantly elevated leaf FA (~55–75%) and sheath FA (~29–72%) in both light conditions. Low light significantly increased the leaf FA in both NT and HL plants; similarly, the sheath FA content was higher under low light, but this was only significant in the HL lines. The line with the highest accumulation of leaf lipids (’HL34’) also had the highest accumulation of Cys-OLE in both light conditions (Table 1 and S2 Fig).

**Table 1:** Effect of low light on total fatty acid and sugars in high lipid Lolium and control.

		Leaves				Sheath		
Genotype	Light	FA (% DW)	Cys-OLE*	HMW (g kg^-1^ DW)	LMW (g kg^-1^ DW)	FA (% DW)	HMW (g kg^-1^ DW)	^1^LMW (g kg^-1^ DW)
NT	Standard	3.69 *F*	nd	63.51 *A*	106.31 *A*	2.17 *F*	99.83 *A*	69.18 *A*
’HL30’	Standard	5.74 *D*	14.33 *D*	62.38 *A*	85.91 *B*	2.80 *E*	102.01 *A*	59.69 *BC*
’HL34’	Standard	6.40 *C*	30.37 *BC*	54.77 *B*	86.48 *B*	3.53 *BC*	96.98 *A*	60.33 *B*
’HL42’	Standard	5.93 *D*	24.53 *C*	54.40 *B*	83.67 *B*	3.05 *D*	105.65 *A*	55.94 *C*
NT	Low	4.22 *E*	nd	1.09 *C*	42.78 *C*	2.25 *F*	6.75 *B*	32.02 *D*
’HL30’	Low	6.91 *B*	39.03 *B*	0.78 *C*	34.51 *CD*	3.15 *D*	6.18 *B*	30.00 *DE*
’HL34’	Low	7.38 *A*	48.54 *A*	0.72 *C*	37.22 *CD*	3.86 *A*	5.84 *B*	28.93 *DE*
’HL42’	Low	6.95 *B*	36.84 *B*	0.60 *C*	33.52 *D*	3.40 *C*	7.39 *B*	27.57 *E*
LSD (95% Confidence)		0.29	8.43	7.71	8.46	0.19	11.78	4.09

^A-F^Means within a column with different alphabets differ (p < 0.05). df = 71 (*n = 9* or *10*).

^1^Data were analyzed using log-transformation.

NT = non-transformed plants; HL = high lipid; FA = fatty acids; DW = dry weight;

Cys-OLE = relative cysteine-oleosin signal intensity detected from immunoblotting (see S2 Fig),

*df = 53 (*n = 10* or *9*).

HMW = high molecular weight sugars; LMW = low molecular weight sugars; nd = not detectable.

Both leaf and sheath WSC concentrations decreased significantly when the plants were grown under low light (Table 1); the decrease was greatest in the HMW-sugar fraction. HMW-sugars accumulated to a higher concentration in the sheath than in the mature leaves; the opposite was true for the LMW- sugar fraction. All plants had lower levels of leaf and sheath HMW-sugars under low light. HMW-sugars were significantly lower in the leaves of two HL plants (’HL34’ and ’HL42’) than the NT under standard lighting. However statistical analysis showed no significant difference of leaf and sheath HMW-sugars between genotypes under low light conditions. In both light conditions, HL leaf and sheath LMW-sugars were lower than NT.

### The influence of light intensity on growth and photosynthesis

**Fig 1:**
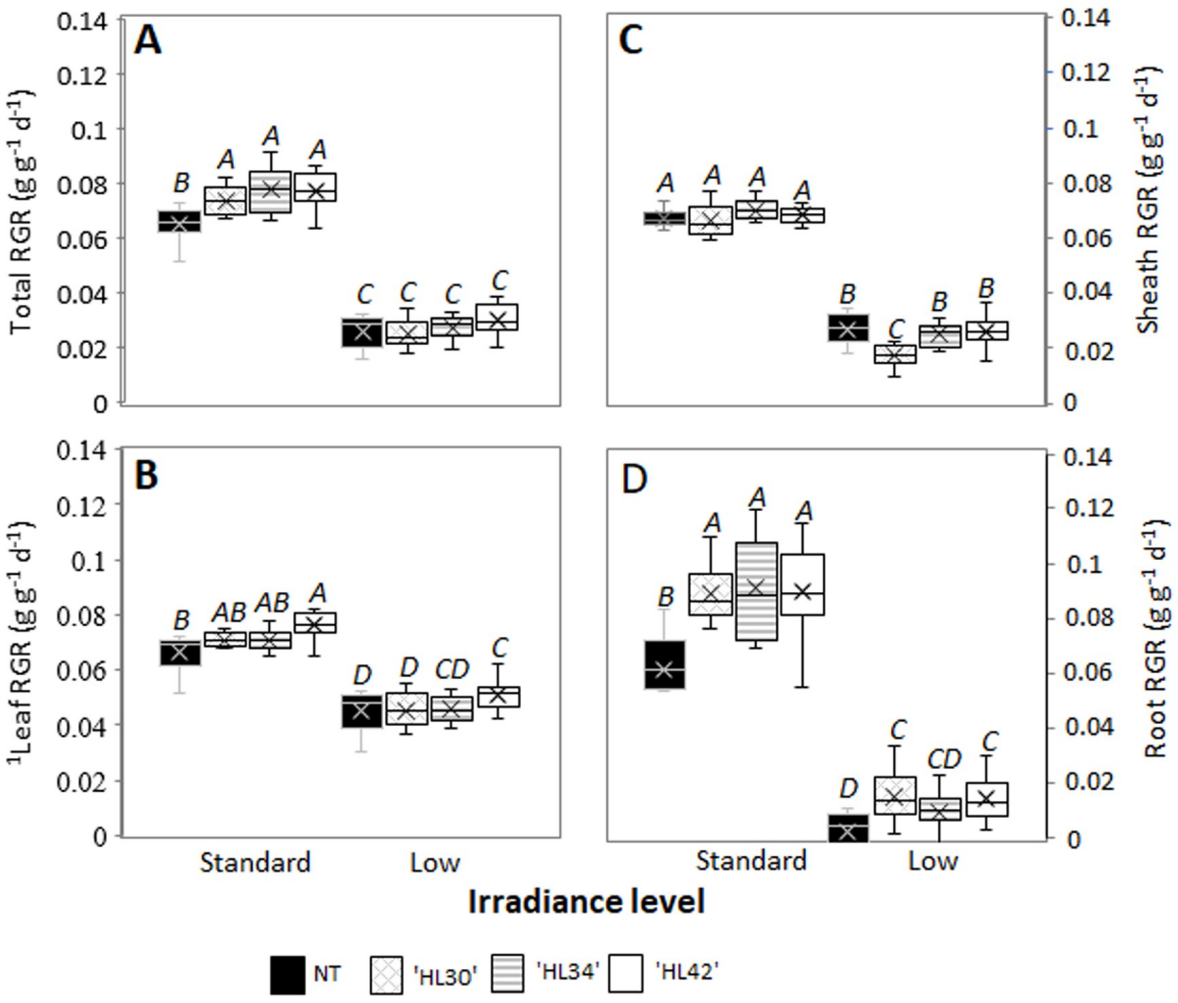
Box and whisker plots of relative growth rate (RGR, g g^-1^d^-1^). **A** RGR of total mass, **B** leaf RGR, **C** sheath RGR and **D** root RGR of high lipid (HL) Lolium and non-transformant (NT) control grown under standard and low lights. ^A-D^Alphabets indicated significant differences (p < 0.05). df = 71 (*n = 9* or *10*). ^1^Data were analyzed using log-transformation. RGR calculated as (ln W_2_ –ln W_1_)/ (t_2_-t_1_), where W_1_ and W_2_ were plant dry weights at times t_1_ and t_2_, respectively.

RGR of all plants grown under standard lighting were significantly greater than plants grown under low light (Fig 1). When comparing plants grown under standard light the overall plant RGR was significantly greater in all HL Lolium compared with NT; the main organs contributing to the elevated RGR in descending order were roots, leaves, and sheath (Fig 1). In contrast, there was no significant difference in the overall RGR of any line when the plants were grown under low light.

HL Lolium had elevated CO_2_ assimilation rates (A), transpiration rates (E), stomatal conductance (*g*_*s*_), electron transport rates (ETR) and quantum efficiency of photosystem II (ɸ_PSII_), compared to NT (Table 2). However, under low light, there were no significant differences in any of these parameters between the HL and NT plants. In all plants the low light treatment significantly reduced A, E, *g*_*s*_, and ETR (55–62%, 49–57%, 47–57%, and 62–64% respectively) while enhancing the ɸ_PSII_ (126–134%) for light-harvesting (Table 2). Under standard light when compared to NT, the HL Lolium have reduced L/R, but increased leaf water and Chl contents. There was significant different in Chl per unit leaf area (Chl_area_) in one of the HL plants (’HL42’). Under low light, L/R, leaf water, and Chl contents increased significantly in both NT and HL plants compared with standard light, the opposite was true for Chl_area_ (Table 2). However, under low light, these parameters did not differ between the NT and HL plants.

**Table 2:** Effect of low light on photosynthesis parameters, leaf-to-root ratio, leaf water, and chlorophyll of high lipid Lolium and control.

Genotype	Light	A^1^ μmol m^-2^ s^-1^	E^1^ mmol m^-2^ s^-1^	*g*_s_^1^ mmol m^-2^ s^-1^	ETR μmol m^-2^ s^-1^	ɸ_PSII_	^1^L/R g g^-1^ DW	Leaf water %	Chl mmol kg^-1^ DW	Chl_area_ mmol m^-2^
NT	Standard	17.97 *C*	1.70 *BC*	224.51 *B*	148.12 *B*	0.50 *C*	0.81 *B*	71.77 *D*	9.59 *D*	0.51 *BC*
’HL30’	Standard	20.87 *AB*	1.96 *AB*	263.98 *AB*	156.92 *A*	0.53 *B*	0.59 *C*	75.47 *C*	10.45 *C*	0.55 *AB*
’HL34’	Standard	19.00 *BC*	1.79 *C*	240.11 *B*	153.32 *AB*	0.52 *BC*	0.53 *C*	75.75 *C*	10.73 *BC*	0.55 *AB*
’HL42’	Standard	22.46 *A*	2.21 *A*	300.20 *A*	158.22 *A*	0.54 *B*	0.59 *C*	77.15 *B*	11.45 *B*	0.59 *A*
NT	Low	8.00 *D*	0.87 *D*	119.21 *C*	56.30 *C*	0.67 *A*	1.85 *A*	83.90 *A*	15.16 *A*	0.47 *CD*
’HL30’	Low	8.52 *D*	0.96 *D*	133.29 *C*	55.60 *C*	0.66 *A*	1.73 *A*	83.92 *A*	14.59 *A*	0.43 *D*
’HL34’	Low	8.73 *D*	0.92 *D*	125.89 *C*	56.98 *C*	0.68 *A*	1.80 *A*	83.87 *A*	15.14 *A*	0.45 *D*
’HL42’	Low	8.54 *D*	0.94 *D*	128.95 *C*	57.35 *C*	0.68 *A*	1.75 *A*	83.84 *A*	14.76 *A*	0.45 *CD*
LSD (95% Confidence)		2.09	0.28	39.40	5.65	0.02	0.21	1.12	0.75	0.05

^A-D^Means within a column with different superscripts differ (p < 0.05). df = 72 (*n = 10*).

^1^Data were analyzed using log-transformation. NT = non-transformed plants; HL = high lipid;

A = the rate of photosynthesis per unit leaf area; E = transpiration rate; *g*_s_ = stomatal conductance; ETR = electron transport rate per unit leaf area;

ɸ_PSII_ = quantum efficiency of PSII; L/R = leaf-to-root ratios; Chl_area_ = chlorophyll per unit of leaf area.

### The influence of light intensity on the P-I curve and photorespiration

P-I curve of HL Lolium was compared with NT plant (S3A Fig). These typical hyperbolic graphs can be characterized by two parameters: the maximum photosynthesis rate (Pm), and the initial slope at low light (α) [48]. The light saturation constant equals the light intensity at the intersection of Pm and α. Photoadaptation factor (Ik) is independent of biomass and is frequently used as a characteristic parameter for comparison of photoadaptation status [49]. Higher Ik was observed in HL Lolium compared with NT plant. Photorespiration measurements showed an increase in the CO_2_ compensation point in the absence of dark respiration of HL plants, and a decreased rate of dark respiration in the light (S3B Fig).

### Transcriptome analysis

A total of 286,962 Trinity transcripts were assembled, with an average contig length of 1,343 bp. The median contig length was 898 bp and the number of total assembled bases was 385,519,512. To reduce the redundancy of the assembly, the two scripts cd-hit-est and tr2aacds were performed. The final assembly contained 51,898 transcripts, with a median contig length of 794 bp and 64,821,560 total assembled bases. BUSCO analysis indicated the final assembly was highly complete, with 87.8% of the complete suite of BUSCO genes identified.

By using Venn plot, we screened for genes commonly differentially regulated in both HL lines when compared to NT plants. It was observed that 3102 genes were commonly downregulated in HL Lolium, and 2898 genes were upregulated (S4A Fig and S1 Data). DEGs affected by the low irradiance growth conditions were identified; 791 commonly downregulated DEGs were observed in NT and both HL lines, while 940 DEGs were commonly upregulated (S4B Fig and S2 Data). To explore the regulatory pathways of these DEGs, GO enrichment analysis was conducted (S4C Fig and S1 Data). A representation of the overall changes in metabolic gene expression in both HL lines relative to NT was also visualized by MapMan software (S5 Fig).

### Genes involved in primary carbon metabolism and photosynthesis

**Fig 2:**
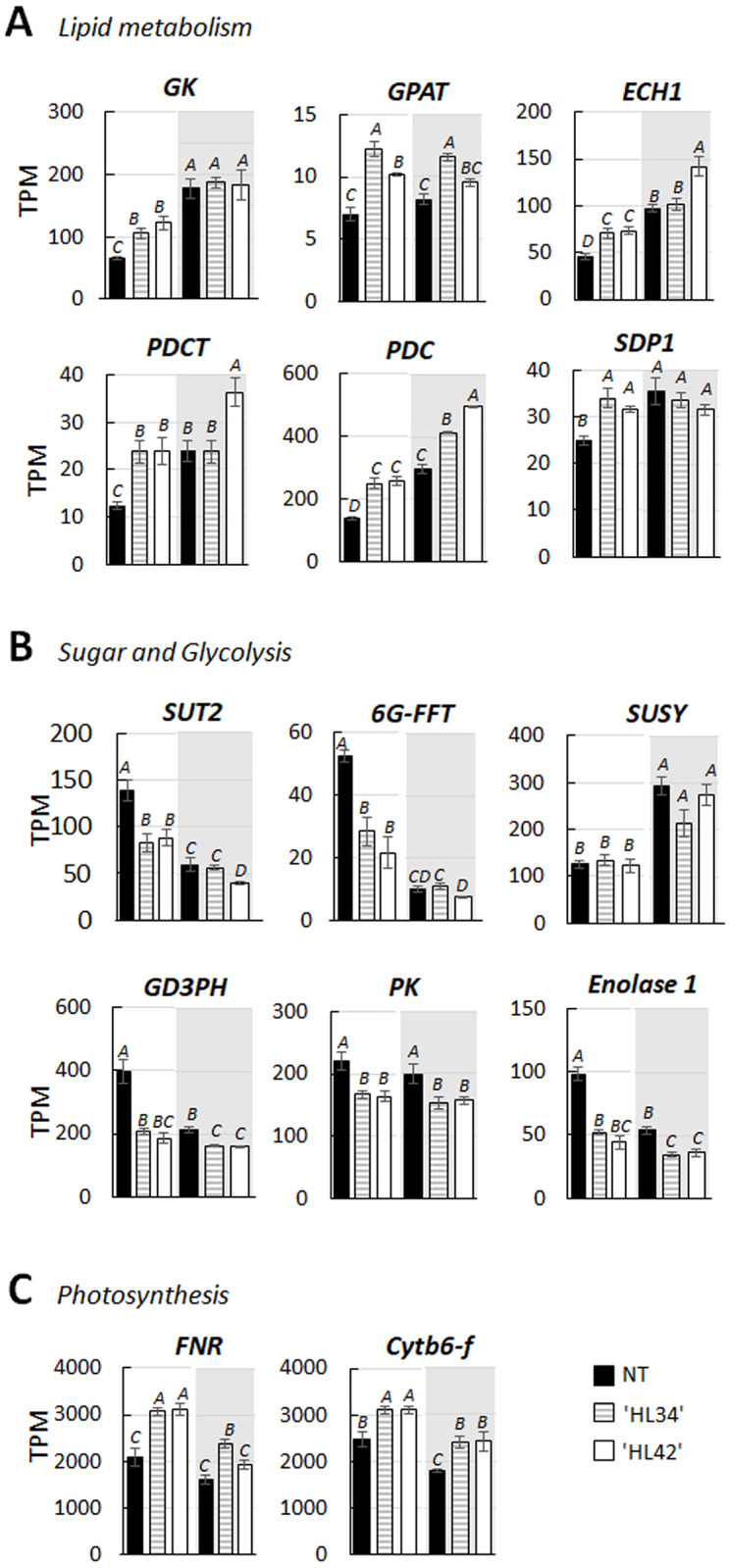
The relative gene expression level of differentially expressed genes (DEGs). Data represent the means of normalized transcript per million (TPM) with the error bar of the SE. DEGs were identified in high lipid (HL) ryegrass compared with non-transformant (NT) plants grown under standard (white graph area) and low (shading graph area) irradiance levels. ^A-D^Alphabets indicated significant differences (p < 0.05, *n = 3*). DEGs grouped using Gene Ontology analysis as following: **A** lipid metabolism: *Glycerol kinase* (*GK*, XP_015636240.1), *glycerol-3-phosphate-2-O-acyltransferase* (*GPAT*, XP_015626270.1), peroxisomal *enoyl-CoA hydratase* (*ECH1*, XP_015632322.1), *phosphatidylcholine*:*diacylglycerol cholinephosphotransferase* (*PDCT*, XP_015644243.1), *pyruvate dehydrogenase* E1 component (*PDC*, XP_015636508.1), and sugar-dependent *triacylglycerol lipase* (*SDP1*, XP_015651228.1), **B** carbohydrate metabolism and glycolysis: *Sucrose transporter protein 2* (*SUT2*, XP_015619709.1), *fructan*:*fructan 1-fructosyltransferase* (*6G-FFT*, XP_015625788.1), *sucrose synthase* (*SUSY*, XP_025882333.1), *glyceraldehyde-3-phosphate dehydrogenase* (*G3PDH*, XP_015625382.1), cytosolic *pyruvate kinase* (PK, XP_015616831.1) and *enolase 1* (XP_015643741.1), **C** photosynthesis pathway: *Ferredoxin NADP reductase* (*FNR*, XP_015640980.1), and *cytochrome b6-f* complex (*Cytb6-f*, XP_015647138.1).

As expected, the recombinant *DGAT* and *Cys-OLE* transcripts were detected at very high levels in HL leaves (approximately log 2-fold change = 18, and 9, respectively, S1 Data) and were not detected in NT. Although the expression of *DGAT1* and *Cys-OLE* were driven by *Rubisco small subunit* and *chlorophyll a/b binding protein* promoters, respectively, transcript levels of these two recombinant proteins were not affected by low light. Overexpression of *DGAT1* and *Cys-OLE* under standard irradiance conditions led to the upregulation of several native genes involved in the initial step in the synthesis of triglycerides, such as *glycerol kinase* and *glycerol-3-phosphate O-acetyltransferase* (*GPAT*), *phosphatidylcholine*:*diacylglycerol choline phosphotransferase 1* (*PDCT*) and TAG catabolism; *peroxisomal enoyl-CoA hydratase 1* (*ECH1*) (Fig 2A and S1 Data). Transcripts of plastidic pyruvate dehydrogenase E1 complex (*PDC*), the enzyme that provides acetyl-CoA and NADH required for *de novo* FA biosynthesis was also increased in HL Lolium. Except for *GPAT*, which was not differentially regulated by low light, the expression of *glycerol kinase*, peroxisomal *ECH1* and *PDCT* were induced in both NT and HL plants grown under low light, whereas the expression levels in NT plant matched to that in the ’HL34’ line. *PDC* transcript levels increased under low light in both NT and HL plants, but there were further significant increases in HL Lolium in this light condition (Fig 2A). Expression of genes involved in TAG catabolism such as *triacylglycerol lipase* sugar-dependent 1 (*SDP1*) and *phospholipase D* were also upregulated in HL Lolium (Fig 2A, S1 and S2 Data). Expression of *SDP1* was increased by low light only in NT whereas the levels were matched to both HL lines.

In this study, DEGs of sugar metabolism and glycolysis were identified. These included *sucrose transport protein 2* (*SUT2*), and fructan biosynthetic gene; *fructan*:*fructan 6G-fructosyltransferase* (*6G-FFT*), which were decreased in HL plants grown under standard light (Fig 2B). These genes were markedly downregulated in both NT and HL plants under low light, whereas a significant difference between genotypes was only detected in standard light conditions (Fig 2B and S2 Data). *Sucrose synthase* (*SUSY*), the *glycosyl transferase* gene encoding the enzyme that catalysed the reversible cleavage of sucrose into fructose and glucose, showed significant upregulation in low light where the expression levels were not affected by genotypes. Genes encoding the glycolytic enzymes such as cytosolic *glyceraldehyde 3-phosphate dehydrogenase* (*GAPDH*), *pyruvate kinase* and *enolase 1* were downregulated in HL Lolium (Fig 2B). Under low light, while transcription of *cytosolic pyruvate kinase* was not significantly influenced, expression of *GAPDH* and *enolase 1* was repressed and showed significant genotype x light interactions (Fig 2B).

GO enrichment analysis showed upregulation of the photosynthesis transcripts in HL Lolium (S4C Fig). These included genes directly involved in light harvesting complexes of photosystems I and II, chlorophyll a/b binding protein, as well as genes involved in the photosynthetic electron transport chain such as *chloroplastic ferredoxin-NADP reductase* (*FNR*) and *cytochrome b6-f* (*Cytb6-f*) complex (Fig 2C, and S1 Data). Under low light, expression of *FNR* and *Cytb6-f* were downregulated in both NT and HL lines. There was significantly higher expression of *Cytb6-f* in HL Lolium, and higher expression of *FNR* in the ’HL34’ line, but not in ’HL42’. (Fig 2C and S2 Data).

### Genes involved in sugar signalling

**Fig 3:**
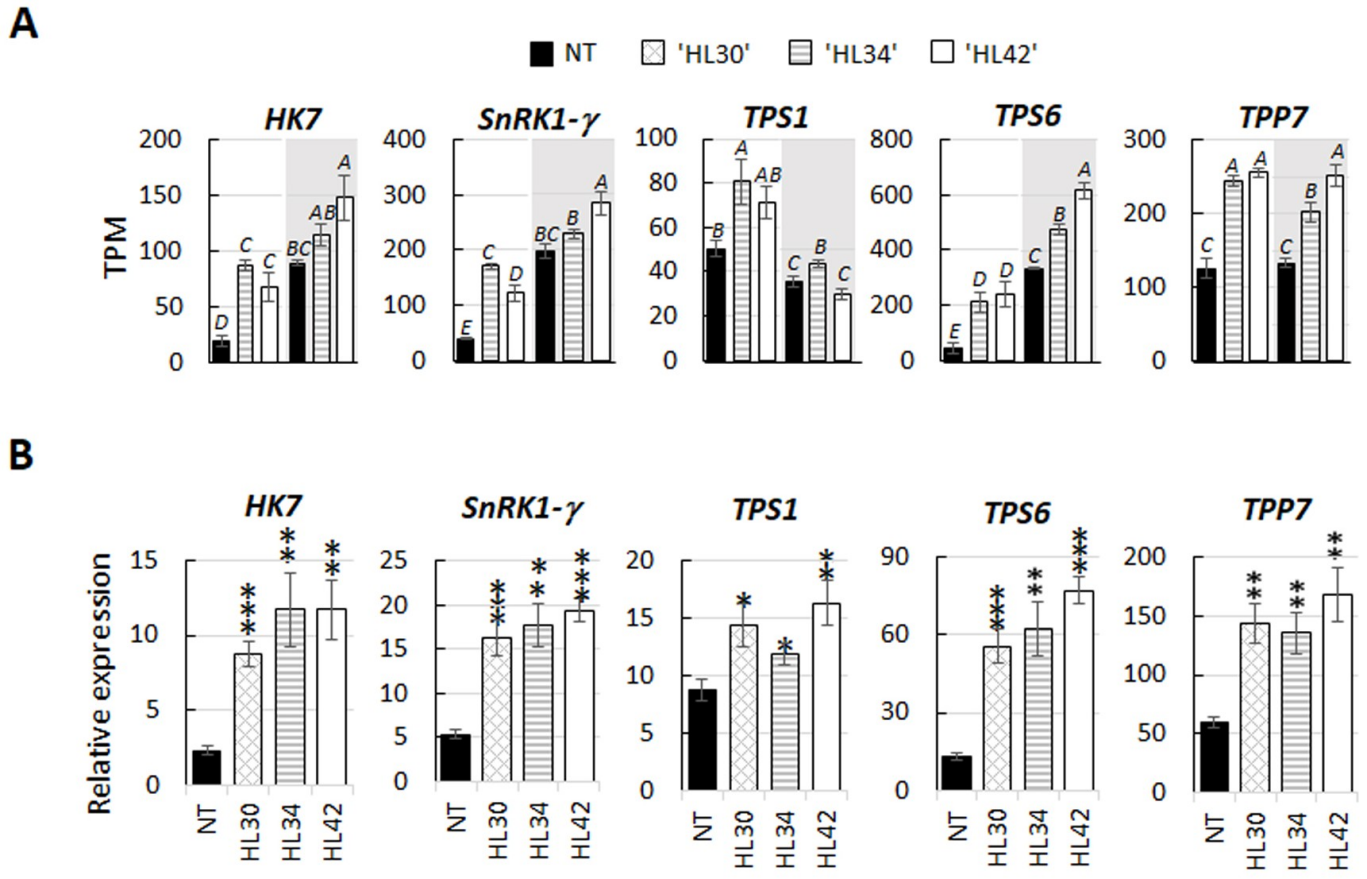
Expression of genes involved in sugar signalling pathway in high lipid (HL) lines. **A** Data represent the means of normalized transcript per million (TPM) with the error bar of the SE, identified in HL Lolium grown under standard (white graph area) and low (shading graph area) irradiance levels, compared to non-transformant (NT) control. ^A-E^Alphabets indicated significant differences (p < 0.05, *n = 3*). **B** Validation of selected genes for relative expression. * indicates p < 0.05, ** for p < 0.01 and *** for p < 0.001 (Student’s t-test). Bars represent the means and SE of five biological replicates. Gene ontology grouped in sugar signalling pathway: *Hexokinase 7* (*HK7*, XP_015637554.1); *sucrose-nonfermented related protein kinase 1 subunit gamma* (*SnRK1*-γ, XP_015635849.1); *trehalose-6-phosphate synthase 1* (*TPS1*, XP_015640390.1); *trehalose 6-phosphate synthase 6* (*TPS6*, XP_015611910.1); *trehalose-phosphate phosphatase 7* (*TPP7*, XP_15651449.1).

**Fig 4:**
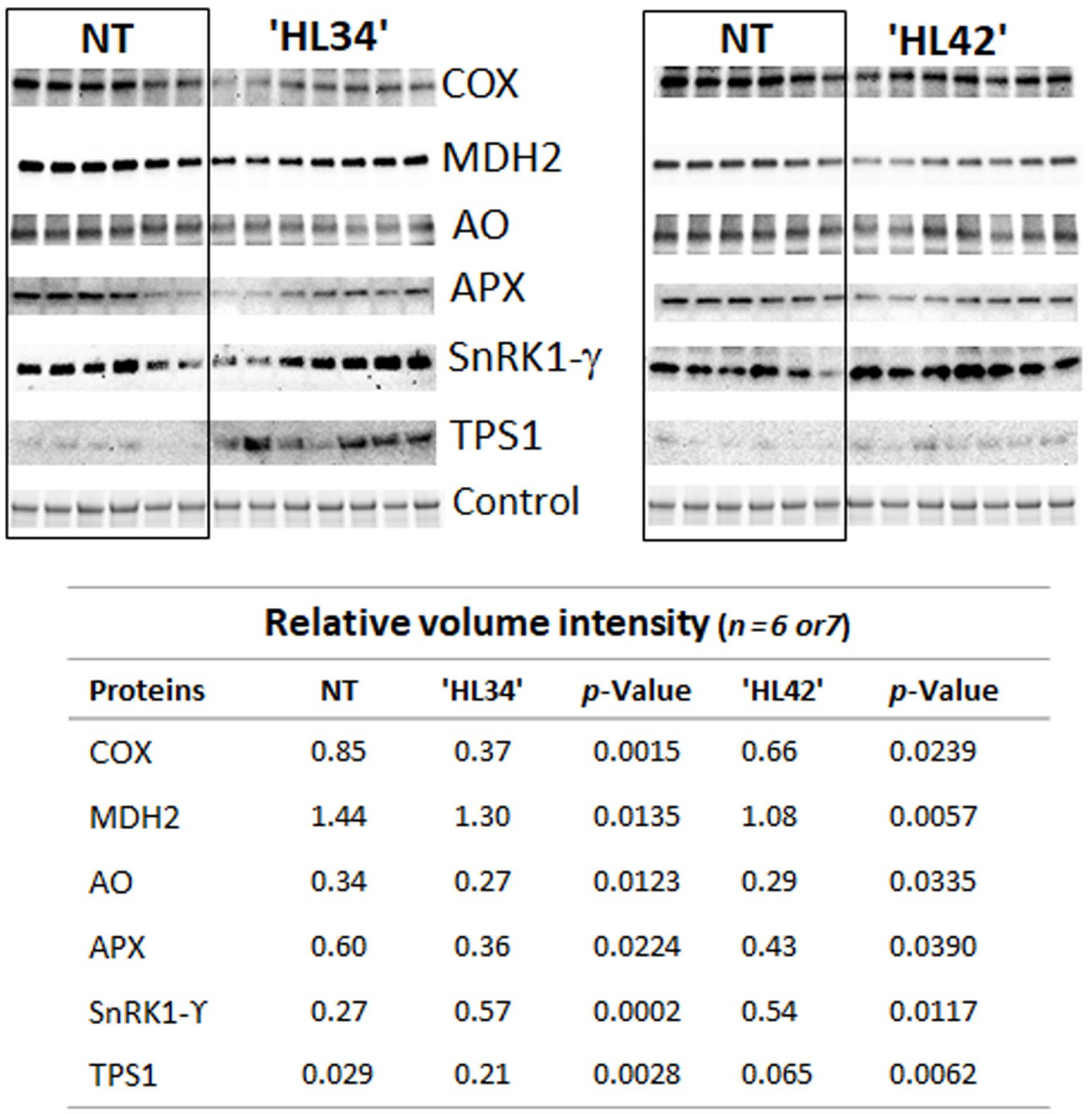
Immunoblot analysis of specific protein abundance in high lipid (HL) Lolium compared with non-transformant (NT) control. Abbreviations: Cytochrome c oxidase (COX, XP_015648280.1); malate dehydrogenase 2 (MDH2, XP_015621604.1); L-ascorbate oxidase (AO, XP_015611052.1); L-ascorbate peroxidase (APX, XP_015630498.1); sucrose-nonfermented related protein kinase 1 subunit gamma (SnRK1-γ, XP_015635849.1); and trehalose-6-phosphate synthase 1 (TPS1, XP_015640390.1).

The majority of differentially related genes involved in sugar homeostasis and signalling such as *hexokinase-7*, *SnRK1* subunit γ (*SnRK1-γ*), trehalose-phosphate synthase 1 & 6 (*TPS1*, *TPS6*) and *trehalose-phosphate phosphatase 7* (*TPP7*) were upregulated in HL Lolium under standard light (Fig 3A). As expected, low light upregulated *hexokinase-7* and *SnRK1-γ* and downregulated *TPS1* transcription in NT. Under low light, the expression of these three sugar signalling components only differed in one of the HL Lolium, compared with the NT plant. In contrast, expression of *TPS6* was significant increased by low light treatment with genotype difference (Fig 3A). Low light did not influence on the expression of *TPP7* in NT, however, one line of HL plants had reduced *TPP7* transcript (Fig 3A). These five selected DEGs of plants grown under standard light were quantitatively analyzed to validate the RNA-Seq data (Fig 3B). Overall, the relative expression trend of these genes agreed with the normalized transcript read. In addition, immunoblot analysis of TPS1 and SnRK1-γ abundance in the leaf protein extraction confirmed the increase of both enzymes in HL Lolium (Fig 4).

### Genes involved in mitochondrial oxidative respiration

**Fig 5:**
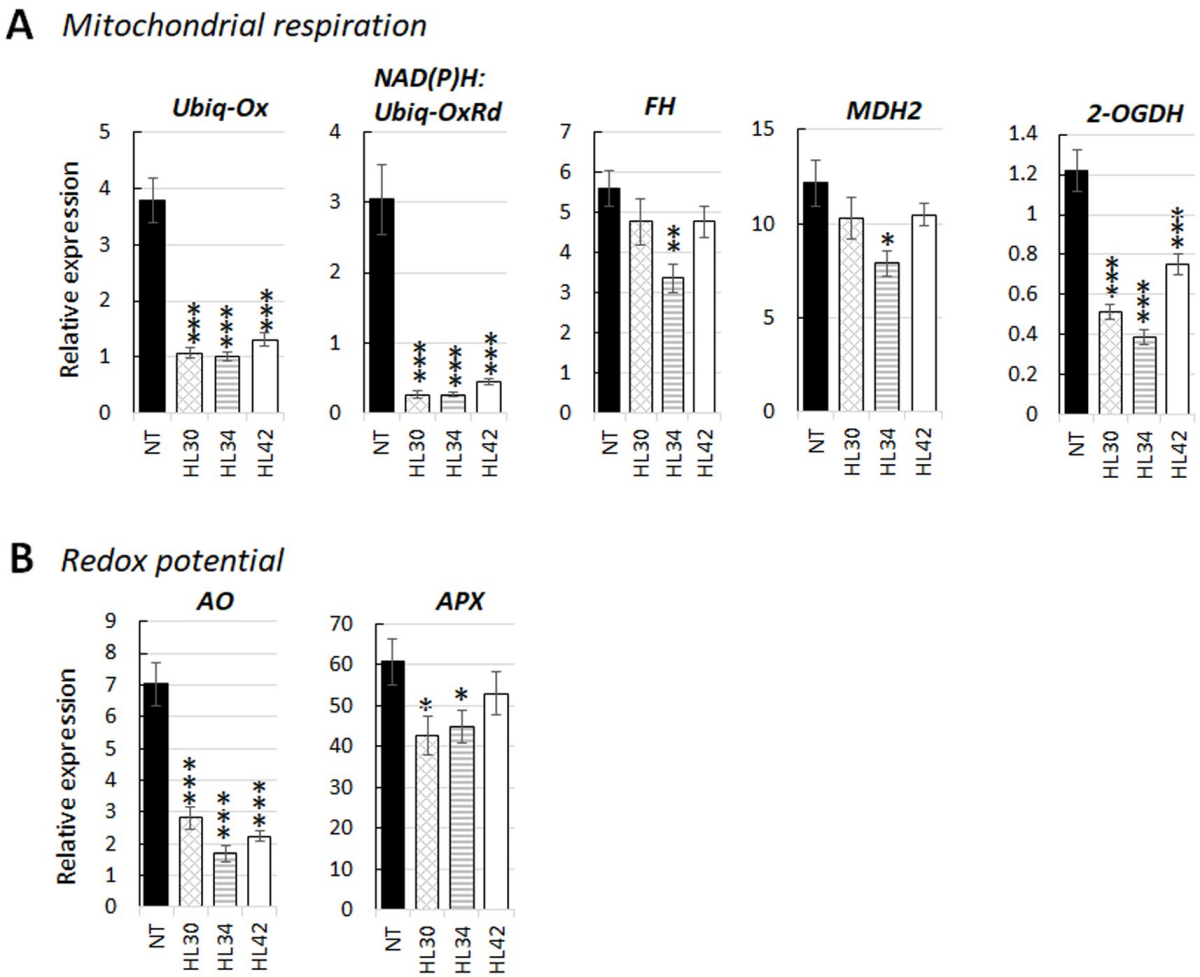
Validation of selected genes in high lipid (HL) lines and non-transformant (NT) plants for relative expression. * indicates p < 0.05, ** for p < 0.01 and *** for p < 0.001 (Student’s t-test). Bars represent the means and SE of five biological replicates. Selected DEGs grouped using Gene Ontology analysis as following: **A** Mitochondrial respiration: *Ubiquinol oxidase* (*UbiQ-Ox*, XP_015635413.1); alternative NAD(P)H: *Ubiquinone oxidoreductase* (*UbiQ-OxRd*, XP_015637913.1); *fumarate hydratase* (*FH*, XP_015633139.1); *malate dehydrogenase 2* (*MDH2*, XP_015621604.1); and *2-oxoglutarate dehydrogenase* (*2-OGDH*, XP_015646480.1). **B** Redox potential: L-*ascorbate oxidase* (*AO*, XP_015611052.1); L-*ascorbate peroxidase* (*APX*, XP_015630498.1).

Under standard light, MapMan software analysis showed downregulation of one single gene in the HL Lolium which was annotated for involvement in the oxidative respiration pathways (S5 Fig). The *de novo* assembly was screened for DEGs of the HL Lolium grown under standard light that related to mitochondrial energy regulation and were shown to be generally repressed, compared with NT plants. These DEGs included mitochondrial *ubiquinol oxidase* (*UbiQ-Ox*), NAD(P)H: *ubiquinone oxidoreductase* (*UbiQ-OxRd*), *cytochrome c oxidase* (*COX*) assembly protein, *fumarate hydratase* (*FH*), *malate dehydrogenase 2* (*MDH2*), and *2-oxoglutarate dehydrogenase* (*2-OGDH*). In NT leaves, these genes were all downregulated by low light (S2 Data and S6 Fig). Under low light, *UbiQ-Ox*, and *UbiQ-OxRd* transcripts were further decreased in HL leaves, and the other four transcript levels did not uniformly significant differ from the NT. Five genes from the above DEGs were selected for qRT-PCR analysis (Fig 5A). Overall, the relative expression trends of these DEGs agreed with the RNA-Seq data, with high significance for the *UbiQ-Ox*, *UbiQ-OxRd*, and *2-OGDH*. Immunoblot analysis of mitochondrial MDH2 and COX subunit II abundance confirmed the decrease in these two enzymes in HL leaf extract (Fig 4).

### Possible changes in leaf redox potential

Under standard light, two cytosolic DEGs, L-*ascorbate oxidase*, and L-*ascorbate peroxidase* transcripts were significantly reduced in HL plants, while the opposite was true for cytosolic *monodehydroascorbate reductase* and respiratory burst *NADPH oxidase* (S1 Data). L-*ascorbate oxidase* and L-*ascorbate peroxidase* were quantitatively analyzed by qRT-PCR to validate the RNA-Seq data. The relative expression trends of these two genes agreed with the normalized transcript read (Fig 5B). Immunoblot analysis of ascorbate oxidase and ascorbate peroxidase abundance confirmed the decrease in these two enzymes in the HL leaves (Fig 4).

### Epicuticular lipid composition

Fatty acyl-CoA reductase enzymes catalyze the reduction of acyl-CoA esters to either aldehydes or alcohol, a key step in the biosynthesis of waxes and wax esters. Preliminary investigation of DEGs involved in the lipid biosynthesis pathways indicated the upregulation of *fatty acyl-CoA reductase* in the HL Lolium by 2–4 fold of the NT plant (S1 Data). This transcriptome result was correlated to our previous studies of the leaf waxes of the HL Lolium by wax composition analysis and SEM. Total wax content (as a percentage of DW) was increased in HL Lolium compared with NT plant (S2 Table). Overall, the altered contents of the HL wax generally changed the portion of relative compositions (when significantly different). There were more the shorter carbon chain of fatty alcohols in the HL epicuticular lipids, while generally the longer chain (C > 27) species did not differ. A decrease in C26 alkane and an increase in C31 and C33 alkanes were observed. There was no difference in aldehydes, FAs, and alkyl esters of HL waxes but a large increase of intracuticular wax; triterpenoid amyrin in HL waxes were detected.

SEM images of leaf adaxial wax crystal were compared between NT and HL plants focussing on high and small dense areas at two different magnifications (S7A and S7B Fig). We observed no significant difference in the size and density of the wax crystal between the plants. There was also no significant difference in the epicuticular wax film observed between these plants (S7C Fig).

## Discussion

Overexpressing of *DGAT1* and *Cys-OLE* promotes carbon flux to FA synthesis in the leaves as evidenced by the increase in several transcripts involved in plastid *de novo* FA biosynthesis. Particularly, there was greater expression of plastidic *PDC* in HL Lolium than in the NT control grown under both light conditions, which contributes to the production of acetyl-CoA building blocks and NADH from pyruvate (Fig 2A) [50]. Expression of peroxisomal *ECH1*, *triacylglycerol lipases SDP1*, and *phospholipases D* is thought to be subsequently influenced by the elevated levels of FA and TAG in HL leaves [10]. This suggests there is a synergistically increased turnover rate of FA and TAG toward β-oxidation, thereby maintaining membrane lipid homeostasis [4]. Under low light, we found no significant change in the level of the recombinant *DGAT1* and *Cys-OLE* transcripts compared with plants grown under standard light (S2 Data). This was interesting given that the *Rubisco small subunit* and *chlorophyll a/b binding protein* promoters used to control the expression of these genes (respectively) can be negatively regulated by low light (S2 Data and reviewed in [51–53]). Moreover, we detected higher Cys-OLE recombinant protein in low light-HL leaves than in the standard light-leaves (Table 1). In consistent, FA in the leaves of low light-grown plants was significantly higher than standard light-grown plants, for both NT and HL Lolium, however, the extent of that increase was significantly higher for HL (17–24%), compared to NT (14%). It remains unknown whether the additional increase is predominantly in the form of TAG or any other lipid classes are included. *DGAT1* expression can be posttranscriptional regulated, whereas OLE expression levels and stability can be associated with histone eviction at the proximal promoters and coding regions and/or posttranslational modification of OLE structure [54–56]. Additionally, several lipogenic enzymes such as fatty acid synthase and acetyl-CoA carboxylase were not significantly differentiated in the HL plants; therefore, the interpretation here may be elusive. Overall, the FA content and transcription profiles of selected genes involved in lipid metabolism are not negatively affected in response to low light. As such, our results support the recent report in [30] that HL Lolium can provide higher lipid and therefore, higher metabolizable energy content, even under light limiting conditions such as pasture sward (shading canopy).

In agreement with greater CO_2_ assimilation and RGR of HL Lolium grown under standard light (Table 2, and Fig 1), the plants appear to have a trend of increased Chl_area_ and ETR, suggesting an increased light-absorption capacity. Consistent with this is the observation of upregulated photosystem I and II-related transcripts of several light-harvesting complexes (S1 Data, and S4C Fig), [57]. In particular, elevated expression of two key photosynthesis regulators: chloroplast *FNR* and *Cytb6 f*, suggests an increase of both linear and cyclic electron flow around photosystem I, a mechanism essential for photosynthesis, in particular the cyclic electron transfer that produces ATP required for driving CO_*2*_ fixation [58,59]. Under low irradiance, plants usually increase their light-harvesting capacity by enhancing the chlorophyll concentration, specific leaf area and membrane lipid content of thylakoid per granum [60,61]. Although this acclimation to different irradiances led to a lower Chl_area_, it improves light transmission allowing higher ɸ_PSII_ and potentially increased photosynthesis by the lower leaves (Table 2, [62,63]). Plastid lipid biosynthesis and the degree of thylakoid stacking were significantly higher in Arabidopsis grown under low light than the plants grown under high light [64]. As such, greater chlorophyll content (Table 2) together with the upregulation of *glycerol kinase* transcript (Fig 2A) and increased leaf FA (Table 1) in both NT and HL plants grown under low light (S2 Data) likely reflect the altered thylakoid ultrastructure of plants that had adapted to this irradiance [65]. Although one of the HL plants had elevated chloroplast *FNR* and *Cytb6-f* transcripts under low light compared with NT, the negative effect of low light on photosynthetic ETR, E, and particularly *g*_*s*_ (Table 2) limits diffusion capacity and therefore constrains CO_2_ assimilation and growth of the plants [66,67]. Although the HL Lolium have greater photosynthetic capacity, this induced phenotype will have a benefit in a field environment as long as other factors e.g. light but also other factors like water and growth temperature are not critical. In plants that photosynthesize more like the HL Lolium, greater transpiration means that more water is lost from the soil because of higher photosynthesis and transpiration, so HL Lolium enter water deficit earlier than NT and photosynthetic rate drops accordingly because of stomatal closure in response to water deficit [32]. HL Lolium tended to perform the best compared to controls in 2019 and 2020 filed trials when rainfall was highest, and the worst compared to controls when rainfall was lowest [30]. In addition, greater water loss in HL Lolium will be exacerbated by higher daytime temperatures over 30 °C. Although this study provides more evidence to support that low light, as usually found in canopy sward conditions, is an unfavourable environmental factor that limits the greater photosynthetic capacity genetically created in the HL Lolium, it has been doubted that low light is the only reason for under performance of the HL Lolium in the tested fields in Missouri [30]. It is possible that in cooler wetter conditions in New Zealand that a yield benefit could still be seen in the field because high photosynthetic capacity would be realised in conditions less susceptible to drought. To test the idea that hot temperature (above 30 °C) and water availability are also a critical factor for the greater growth of HL Lolium, irrigated swards with different temperature controls could be investigated.

Under standard light, HL Lolium have greater leaf water content although the higher transpiration rate was detected (Table 2). It will be interesting to see if there is altered non-stomatal transpiration. In this study, we identified the increased transcripts of fatty acyl-CoA reductase, an enzyme involved in the biosynthesis of waxes and wax esters, and significant increase in total wax and wax content of triterpenoid, β-amyrin (S2 Table); albeit no change in the size and density of wax crystals on the HL leaf surface was observed (S7 Fig). *In vitro*, mouse DGAT1 exhibits additional acyltransferase which catalyzes the synthesis of wax esters [68]. In fact, a large number of wax ester synthase/DGAT1 homologs was identified in plants, several were characterized as functioning in cuticular wax synthesis [69,70]. In addition to the DGAT1 role in TAG synthesis [10,68], we speculated that overexpression of DGAT1 in HL Lolium affects content of other different lipid classes, including wax esters, either direct or indirect pathways. Wax triterpenoids have been shown to provide mechanical and thermal stability to the plant cuticle; additionally, some reports suggested triterpenoids play no or only a minor role as transpiration barriers [71]. Further studies on the influence of wax β-amyrin on HL Lolium transpiration may be interesting.

Ryegrass stores very little starch: most photosynthetically fixed carbon is directed into sucrose and fructan (HMW-sugars) synthesis [72,73]. Increased lipid accumulation in the vegetative tissues could alter carbohydrates metabolism, as supported by the observed reduction of plant sugar content [10,11,74]. Although the levels of LMW-sugars in leaf and sheath of HL Lolium were significantly lower than NT, we did not observe a significant change of transcripts encoding of enzymes involved in sucrose synthesis and cleavage but only the lower transcript of *SUT2* (Fig 2B) which suggests plant cells adjust their transport of sucrose [74]. The decline of 6G-FFT transcripts correlates with the lower HMW-sugars in HL leaves. A major reduction of *SUT2* and *6G-FFT* transcripts was further observed in both NT and HL Lolium grown under low light. This also correlated with the substantially lower levels of WSC in the leaves and sheaths under low light (Table 1). Given that low light limits carbon capture of the plants and reduces sugar available for transportation through the plant cells, it is unsurprising to see a significant reduction of biosynthesis and storage of fructan, which usually occurs only when carbon assimilation exceeds carbon use [72].

Sugars play a role in the feedback inhibition of photosynthesis through hexokinase and SnRK1/T6P signalling pathways; the processes have been well-known as the primary components of sugar homeostasis in plants [16,75]. It has been speculated that the trade-off between lipids and sugars in high lipid leaves may alter the leaf sugar homeostasis which in turn mitigates negative feedback on photosynthesis [13]. In this study, we observed an increase in the expression of these three sugar sensing regulators (Fig 3). Hexokinase 7 is a multifunctional enzyme, regulating glycolytic flux in the cytosol and acting as a glucose sensor in the nucleus [76,77]. The elevation of hexokinase 7 in the HL Lolium may be to promote more substrate for the oxidative pentose phosphate pathway and subsequent NADPH production which is required for *de novo* FA synthesis. SnRK1 is a central regulator in adjusting cellular energy and metabolism during starvation, stress and growth-promoting conditions [78,79]. With the level of low sugars available in HL Lolium, the upregulation of SnRK1-γ in these plants is unsurprising given there is a shift in the level and the form of primary energy [62]. Recent work by Zhai et al. [80] demonstrated that T6P can regulate FA synthesis by antagonistically regulating SnRK1 activity through binding directly to the SnRK1, blocking interaction to an activator kinase, thereby diminishing the activity of SnRK1. This study suggests that switching the destination of fixed carbon and energy form from sugars to FA in source organs may perturb other unidentified intermediary factors that link a complex interaction between sugar signalling and FA homeostasis. T6P level is positively regulated by sucrose status, by activating TPS1 and/or inhibiting [81]. Increased TPS1&6 and TPP7 transcript levels in HL Lolium suggests the direction of trehalose metabolism toward the intermediate T6P, followed by synthesizing of the end-product trehalose. Although there is increasing evidence for a differentiate role between T6P as a sucrose signal and trehalose as stress response, a regulator of stomatal conductance and water-use efficiency, our understanding of the sucrose-T6P-trehalose nexus is still fundamental [81,82]. Overall, gene expression and protein level studies may not be sufficiently relevant readout of trehalose/T6P/SnRK1 complex activity that is modulated in high lipid accumulating photosynthetic tissues. To elucidate how trehalose and/or T6P is integrated with the lipid metabolism and the modes of action, detailed analyses of metabolic levels and fluxes are essential and in continuing in our studies.

Boosting the production of FA in HL leaves should promote the consumption of reducing power [23]. Here, we hypothesized that the additional requirement of reductant is probably satisfied by reducing non-photochemical quenching [83]. This is consistent to the observation of higher ɸ_PSII_ in HL Lolium. The greater demand of energy for *de novo* lipid biosynthesis is probably achieved by reducing the amount of energy diverted to xanthophyll cycle thermal energy dissipation, thus, increased PSII efficiency [25]. Our speculation is related to a higher photoadaptation factor of the P-I curve and CO_2_ compensation point which coincides with decreased dark respiration in the light in HL Lolium (S3 Fig) shown in this study and in [10]. Respiration removes excess carbohydrates when the storage pool becomes full, therefore, a reduction of sugars in HL leaves means less respiration [15]. The reduced levels of cytosolic *G3PDH*, *pyruvate kinase* and *enolase 1* transcripts in HL Lolium suggests changes in glycolytic enzyme activities due to availability of sugars and relates closely to respiration rate [84]. Thus, repression of several transcript levels of genes encoding mitochondrial oxidative respiratory enzymes such as UbiQ-Ox, alternative Ubiq-OxRd, COX, MDH2 and 2-OGDH in the HL leaves (Fig 5A) can be simply explained by this way. Another explanation is that additional demand for energy and reductants in HL Lolium reduces the requirement of mitochondrial energy dissipation for reducing excess redox equivalents [25]. Generally, plant cells export excess reductants out of the chloroplast as malate through the "malate valve", where it can be consumed by malate dehydrogenases in the cytosol and peroxisomes or directly used through the mitochondrial TCA cycle [85]. Under these premises, our understanding and assessment for certain energy conversion processes appears to be a new perspective.

The HL Lolium also differentially regulated the transcripts of enzymes involved in redox homeostasis. Two cytosolic enzymes: L-ascorbate oxidase and L-ascorbate peroxidase, were identified as significantly reduced in HL plants (Fig 4). The opposite was true for the cytosolic *monodehydroascorbate reductase* and respiratory burst *NADPH oxidase* transcripts (S1 Data). L-ascorbate oxidase has a prominent role in redox homeostasis; therefore, it may be necessary for the HL Lolium to manipulate the level of this enzyme to maintain their redox balance [86]. As mentioned, promoting the production of FA requires reducing components like NADH or NADPH. We speculate that decreased levels of L-*ascorbate oxidase* (thus increased levels of reduced form-ascorbate) may help reduce the recycling process of ascorbate from oxidized forms (dehydroascorbate and monodehydroascorbate) and in doing so reserve more reduced forms of glutathione and NADPH [87]. Similarly, a reduction of L-*ascorbate peroxidase*, an increase in *monodehydroascorbate reductase*, and a synergistic activation of *NADPH oxidase* transcripts should lead to the preservation of more reduced ascorbate form and a direct connection to reactive oxygen species [88,89]. We hypothesize that HL Lolium may maintain their intracellular ascorbate redox state mainly in the reduced state in response to their oxidative stress, caused by the greater use of reducing power for FA biosynthesis under normal light and/or the crossed-linking of recombinant Cys-OLE expression for lipid accumulation [3,90,91]. Given that the mechanisms in the ascorbate-glutathione cycle are complex with different isoforms classified from multigenic families and can be regulated by multiple factors [92,93], the results here are preliminary and further experiments will be required to thoroughly determine the change in redox potential in high lipid plants.

## Conclusion

Our previous studies reported that creating stable lipid droplets within the photosynthetic cells of the HL Lolium reduces the availability of primary photosynthate (sugars). In turn, this reduces the diurnal build-up of carbohydrates and prevents feedback inhibition on photosynthesis. This study reports changes in the expression level of numerous genes when carbon is diverted to the accumulation of lipids in their leaves. Many upregulated transcripts are involved in lipid metabolism, light-capturing, and photosynthesis. The opposite was true for sugar biosynthesis and transportation. These were also shown to be modulated by the low intensity of the photosynthetic light. The results from this study suggest that development of the HL Lolium could provide a promising option towards delivering pasture sward (low light field canopy) with higher lipid content. However, the secondary effect of the HL expression that increases photosynthesis and growth yield of the plants may be not efficiently translated from the spaced pots to sward canopy, due to shading conditions. Some management practices may be required to improve the growth benefit of HL ryegrass in the field under grazing conditions while other critical factors of plant growth such as temperature and water should not be excluded.

In addition, we also reported that the expression of genes involved in sugar signalling, mitochondrial oxidative phosphorylation, and redox homoeostasis were all influenced by the accumulation of lipid in the leaf. These results emphasize the importance and complexity of the regulatory mechanisms impacted by perturbing the primary carbon metabolism. Further detailed studies on the redox potential in HL plants in relation to their response to other environmental stresses such as drought or salinity are ongoing.

## Supporting information

S1 Fig The circadian rhythm of lipids and sugars in high lipid (HL) Lolium and segregated null control. **A** Light/dark cycle for the plant growth and harvesting hours. **B** The plant ramets were harvested and divided into 5 organs: Leaf tip, leaf middle, ligule, sheath and root. **C** Diurnal changes in the concentration of lipids. Asterisks indicate statistically significant difference between the end of day and night of each genotype based on Student’s t-test (*n = 5*, ** for p < 0.01 and *** for p < 0.001). **D** Diurnal changes in the concentration of low molecular weight (LMW) and high molecular weight (HMW) sugars. Asterisks indicate statistically significant difference between NT and HL for p < 0.05.
(JPG)

S2 Fig Determination of cysteine-oleosin (Cys-OLE) accumulation in leaves. **A** Stain-free gel analysis of total proteins extracted from 25 mg of leaf dry material of high lipid (HL) Lolium and non-transformed plants (NT) grown under low and standard lights. Images showed ~56 kDa band intensity visualized from stain-free gels. **B** Immunoblotting results using anti-oleosin antibody. **C** Box and Whisker plots of volume intensity of total proteins. **D** Cys-OLE. **E** Normalized volume intensity of Cys-OLE to total proteins.
Samples 1–10 represent the 10 biological replicate plants. C1 and C2 were controls of protein loading and immunoblotting references. ^A-D^Alphabets indicated statistical difference (p < 0.05). *n = 9* or *10*, df = 53.
(JPG)

S3 Fig Measurement of photosynthesis parameters. **A** Photosynthesis-irradiance curve showing the three normalized photophysiological parameters, the maximum photosynthesis rate Pm, the initial slope α, and the photoadaptation factor Ik of high lipid Loilum (’DGAT1+CO4’ and ’DGAT1+CO5’, [12]) compared with non-transformant control (NT). **B** Photorespiration measurement showing the CO_2_ compensation point in the absence of dark respiration г*, and the rate of dark respiration in the light Rd.
(JPG)

S4 Fig Transcriptome analysis of differentially expressed genes (DEGs). Venn plot showing the overlap of the number of significant DEGs which were downregulated (blue) or upregulated (red) in high lipid HL Lolium, compared to non-transformant (NT) control. **A** Comparison analysis of plants grown under standard light (STD). **B** Comparison analysis of plants grown under standard and low light (LOW). Numbers represent differentially expressed gene numbers. **C** GO analysis of DEGs. The bubble size represents gene numbers in each regulatory pathway and the colour gradient represents the log10-fold difference.
(JPG)

S5 Fig MapMan analysis of the metabolic pathways of differentially expressed genes (DEGs). Each inset presents a DEG between high lipid Lolium and the non-transformant control. The red lattice represents upregulated genes, and the blue lattice represents downregulated genes. The colour scale presents the log 2-fold change value of DEGs.
(JPG)

S6 Fig The selected gene expression level of high lipid (HL) Lolium. Plants included non-transformed (NT) control were grown under standard (white graph area) and low (shading graph area) irradiance levels. Data represent the means of normalized transcript per million (TPM) with the error bar of the SE. ^A-D^Alphabets indicated significant differences (p < 0.05, *n = 3*).
(JPG)

S7 Fig SEM analysis of leaf adaxial wax. Wax crystal were compared between non-transformant control (NT) and high lipid (HL) Lolium. **A** Images were taken from the high dense area with two different magnifications. **B** Images from less dense areas. **C** Epicuticular wax film comparisons.
(JPG)

S1 Table List of primers and their respective sequences for quantitative RT-PCR of perennial ryegrass genes. Locus identifier indicates the best-hit identification of the differentially expressed contigs from BLAST searches of the Oryza sativa IRGSP-1.0 databases. Lp-elF-4α and Lp-TEF1 were used as reference genes in this study.
(DOCX)

S2 Table Composition of wax compounds in high-lipid Lolium (HL) and non-transformant control (NT). (DOCX)

S1 Data Transcriptome analysis of differential gene expressions in high lipid Lolium. (XLSX)

S2 Data Transcriptome analysis of differential gene expression in Lolium under different irradiance. (XLSX)

S1 Protocol Experiments, plant establishment, growth, and harvest of high lipid (HL) Lolium for diurnal sugars and FA analysis. The diurnal experiment was conducted with ten clonal plantlets each of five segregation progenies of T2 homozygous HL Lolium and five segregated null plants (cultivar ’Alto’ x ’Elite 50’, PGG Wrightson, NZ). Homozygous HL Lolium with ‘Alto’ background were recently generated through a commercial breeding programme and used for field trials in the Midwest of the United States of America [30]. Therefore, this supplementary experiment was conducted using the ‘Alto’ lines as ‘preferred field-tested material’ than the previous generated HL Lolium with ‘Impact’ background (laboratory-tested material). After the last synchronization round, plants were grown through an ’establishment period’ in washed coarse sand (as described in the main experiments) in a controlled environment room with standard light. At the end of this ’establishment period’, all plantlets were defoliated to 5–6 cm above the sand surface and regrown for an additional three weeks with similar treatment as described in the main experiments under standard light. Plants were destructively harvested at the start of daylight (0 h) through the next day (24 h) with 4 h time-point intervals (S1A Fig) and divided into 5 organs (S1B Fig): Leaf tip, leaf middle, ligule, sheath and root (cleaned). Plant materials were freeze-dried for 3–4 d and stored at -80 °C until use for sugars and FA analysis (S1C and S1D Fig).
(PDF)
