# Supplementary figures and images for: Insight into the regulatory networks underlying the high lipid perennial ryegrass growth under different irradiances
Source: PLoS One. 2022 Oct 13;17(10):e0275503. doi: 10.1371/journal.pone.0275503 (PMC9560171; doi:10.1371/journal.pone.0275503)

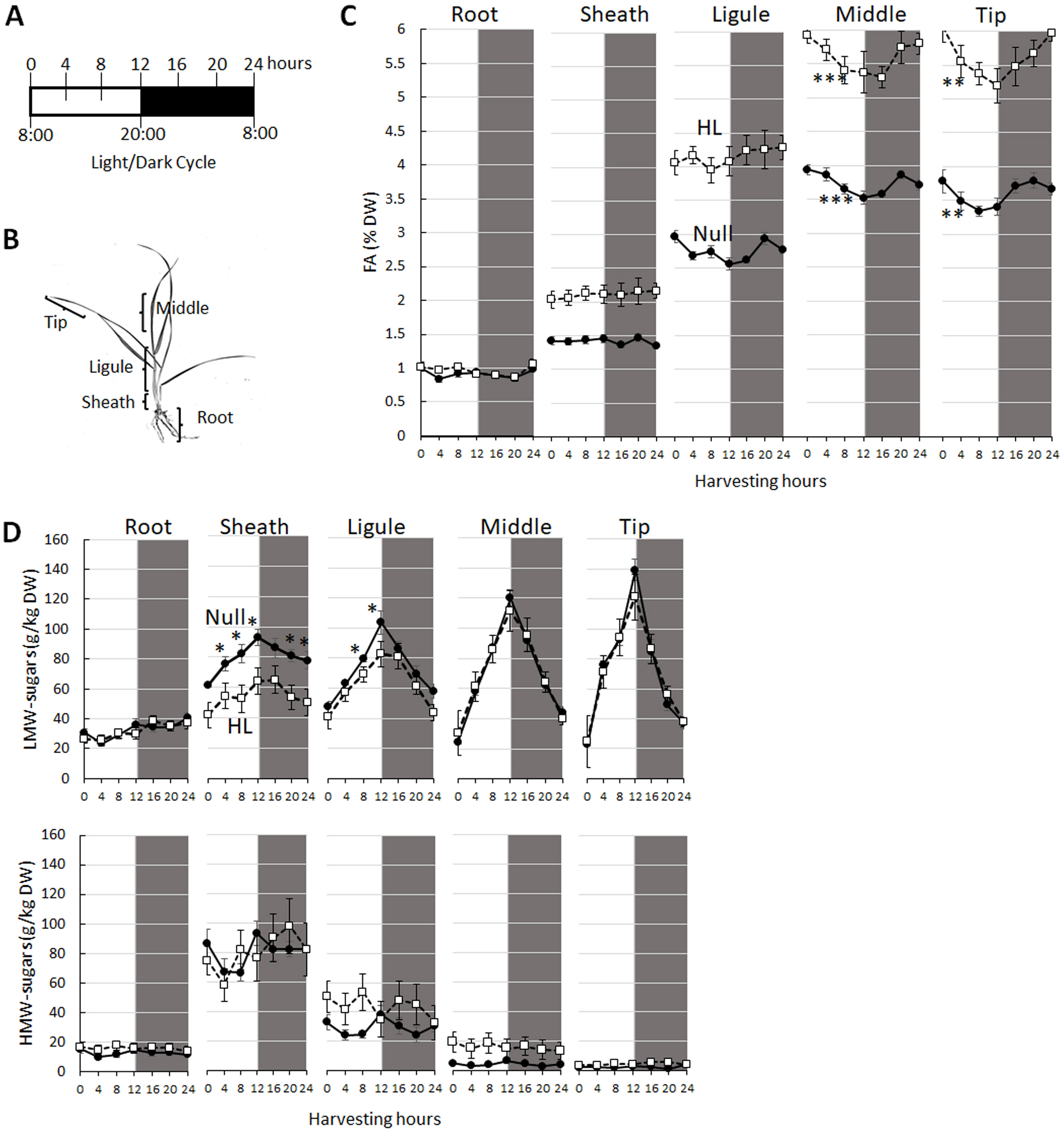

Supplement: S1 Fig — A Light/dark cycle for the plant growth and harvesting hours. B The plant ramets were harvested and divided into 5 organs: Leaf tip, leaf middle, ligule, sheath and root. C Diurnal changes in the concentration of lipids. Asterisks indicate statistically significant difference between the end of day and night of each genotype based on Student’s t-test (n = 5, ** for p < 0.01 and *** for p < 0.001). D Diurnal changes in the concentration of low molecular weight (LMW) and high molecular weight (HMW) sugars. Asterisks indicate statistically significant difference between NT and HL for p < 0.05. (JPG) [file pone.0275503.s001.jpg]

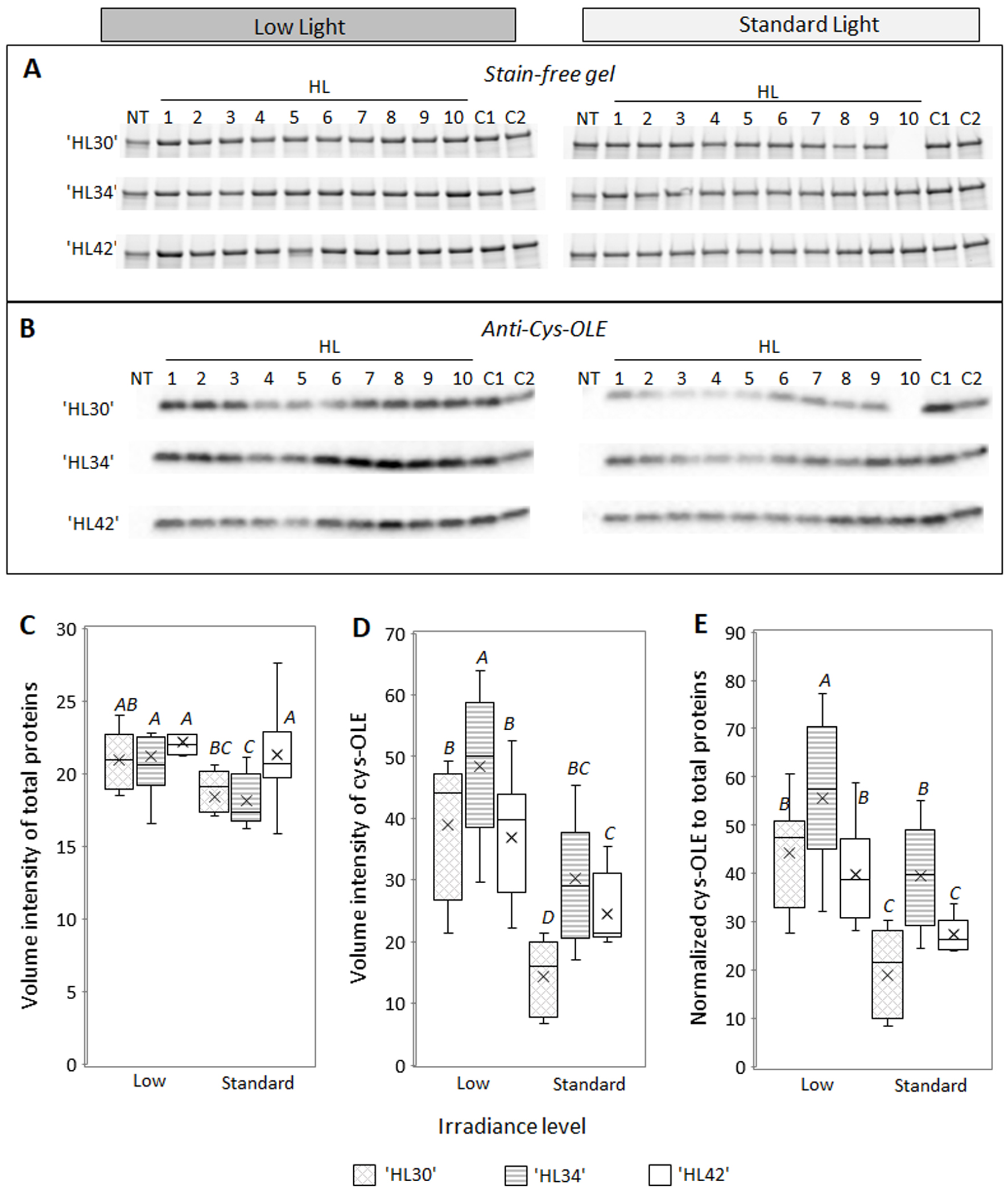

Supplement: S2 Fig — A Stain-free gel analysis of total proteins extracted from 25 mg of leaf dry material of high lipid (HL) Lolium and non-transformed plants (NT) grown under low and standard lights. Images showed ~56 kDa band intensity visualized from stain-free gels. B Immunoblotting results using anti-oleosin antibody. C Box and Whisker plots of volume intensity of total proteins. D Cys-OLE. E Normalized volume intensity of Cys-OLE to total proteins. Samples 1–10 represent the 10 biological replicate plants. C1 and C2 were controls of protein loading and immunoblotting references. A-DAlphabets indicated statistical difference (p < 0.05). n = 9 or 10, df = 53. (JPG) [file pone.0275503.s002.jpg]

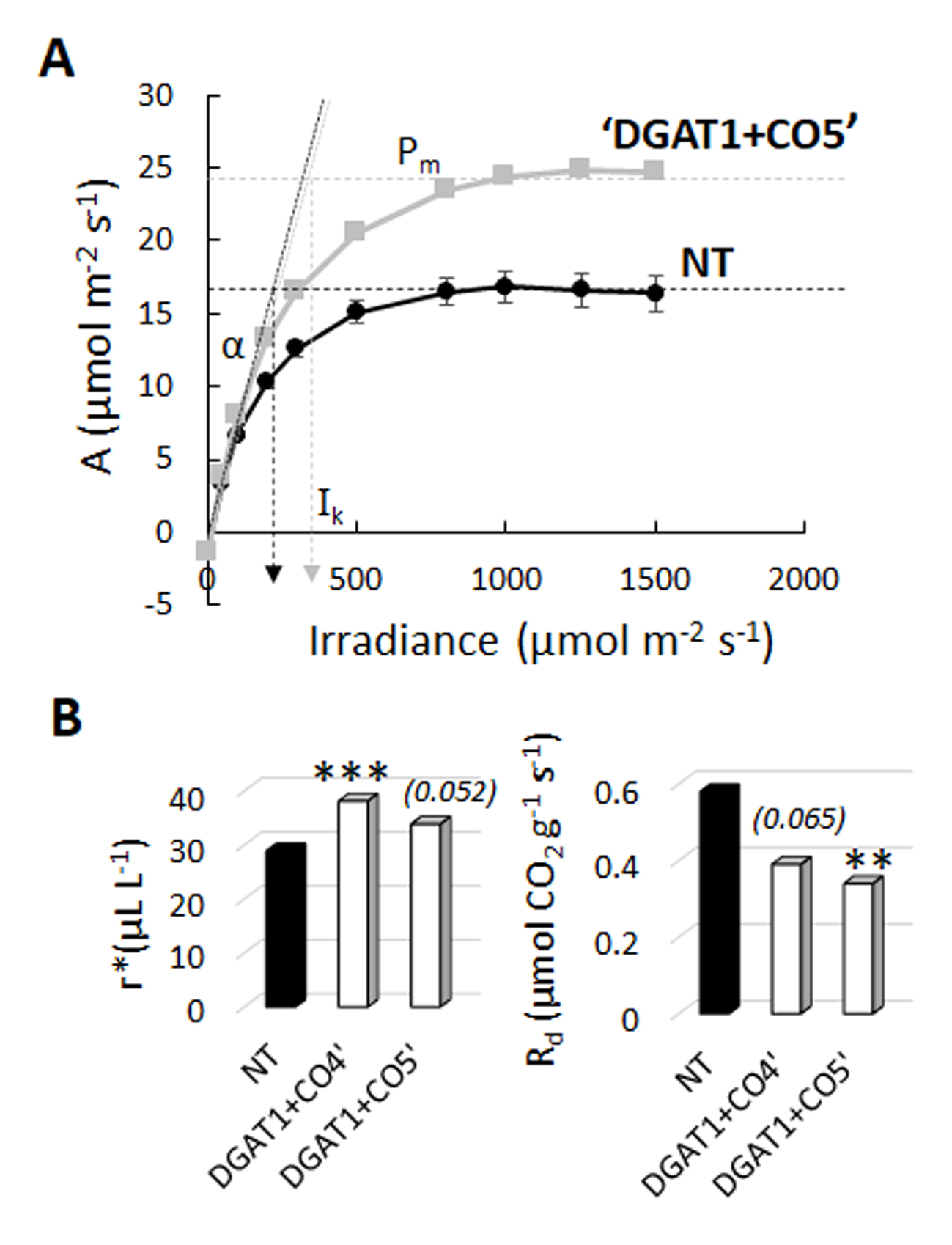

Supplement: S3 Fig — A Photosynthesis-irradiance curve showing the three normalized photophysiological parameters, the maximum photosynthesis rate Pm, the initial slope α, and the photoadaptation factor Ik of high lipid Loilum (’DGAT1+CO4’ and ’DGAT1+CO5’, [12]) compared with non-transformant control (NT). B Photorespiration measurement showing the CO2 compensation point in the absence of dark respiration г*, and the rate of dark respiration in the light Rd. (JPG) [file pone.0275503.s003.jpg]

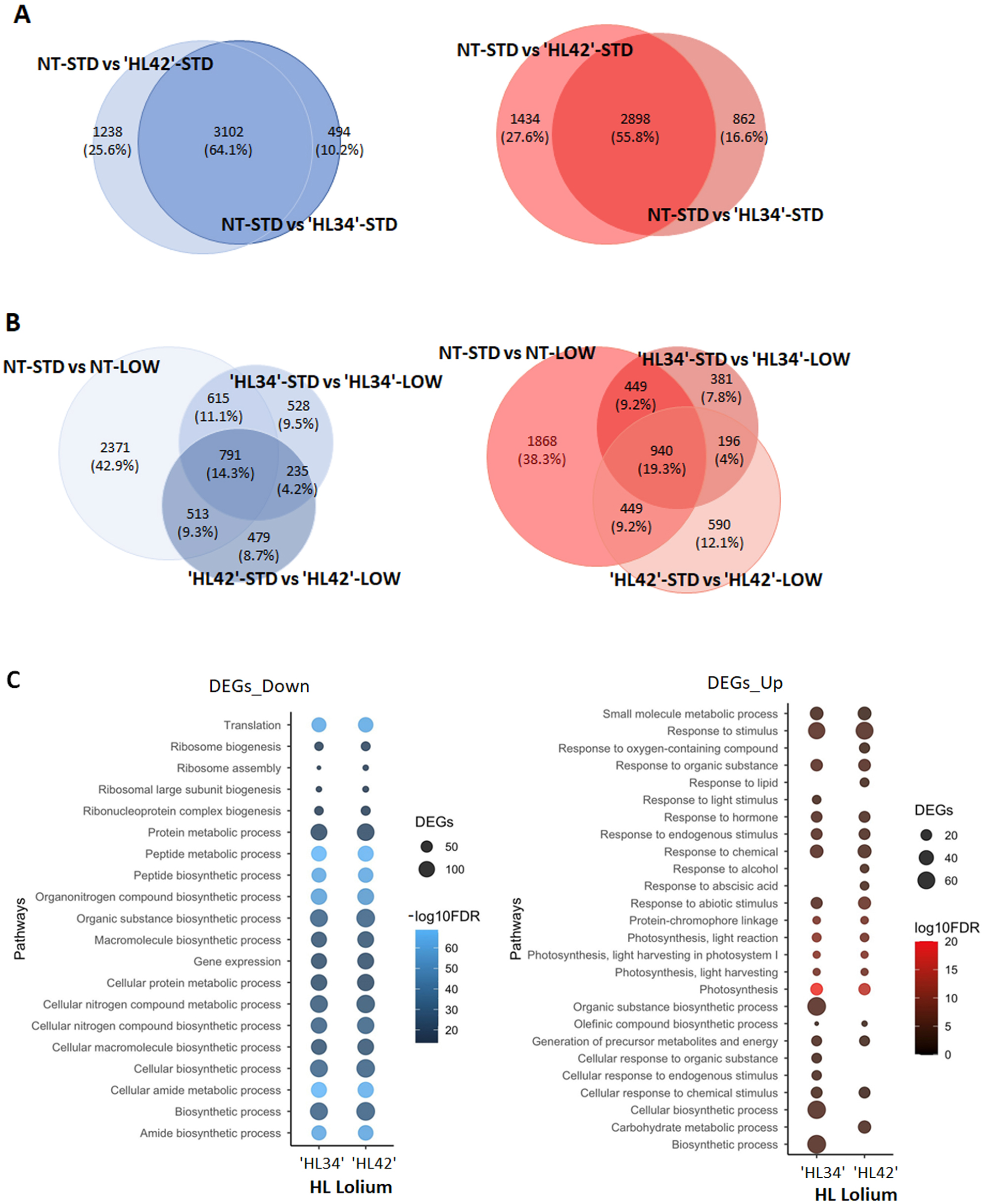

Supplement: S4 Fig — Venn plot showing the overlap of the number of significant DEGs which were downregulated (blue) or upregulated (red) in high lipid HL Lolium, compared to non-transformant (NT) control. A Comparison analysis of plants grown under standard light (STD). B Comparison analysis of plants grown under standard and low light (LOW). Numbers represent differentially expressed gene numbers. C GO analysis of DEGs. The bubble size represents gene numbers in each regulatory pathway and the colour gradient represents the log10-fold difference. (JPG) [file pone.0275503.s004.jpg]

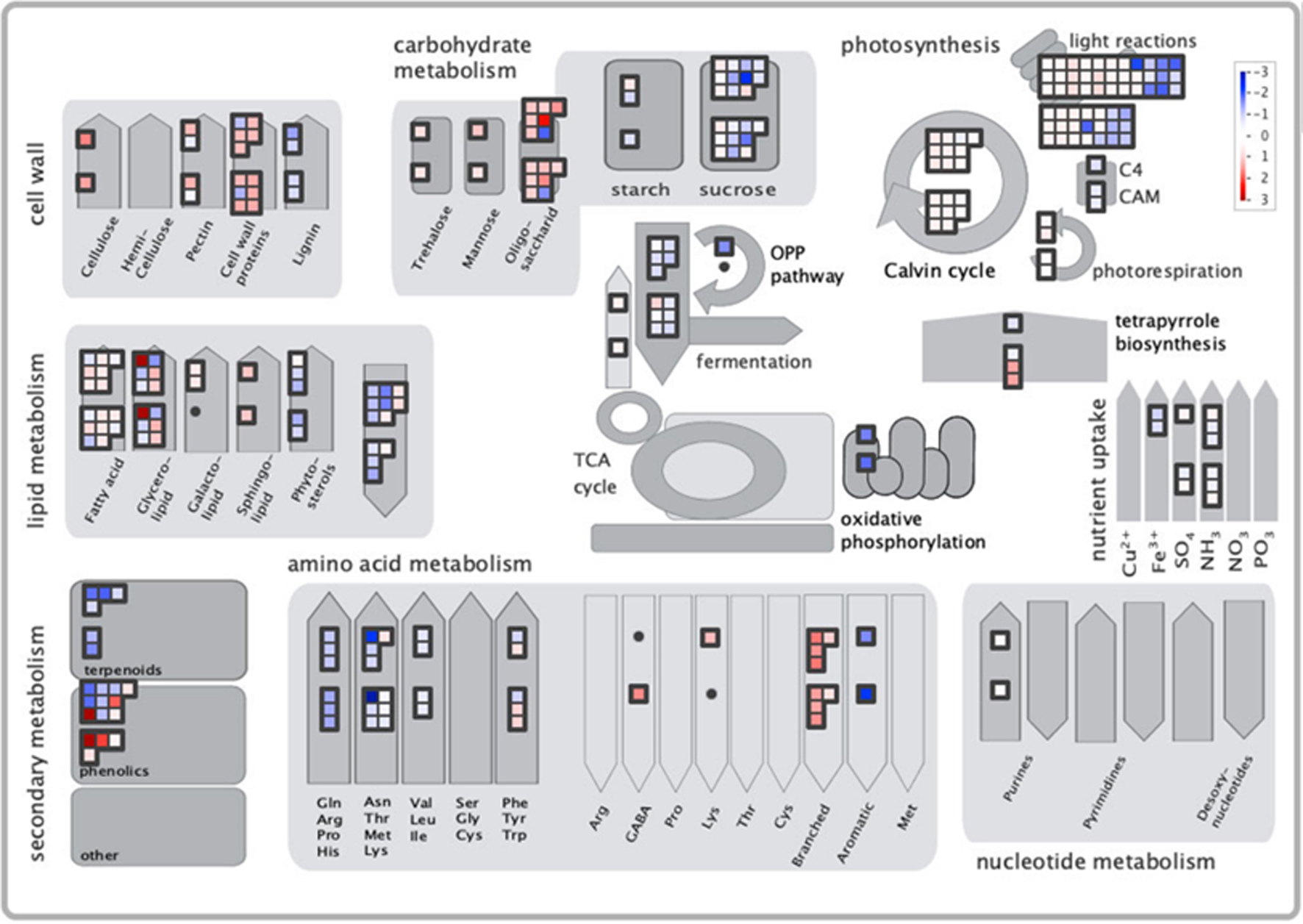

Supplement: S5 Fig — Each inset presents a DEG between high lipid Lolium and the non-transformant control. The red lattice represents upregulated genes, and the blue lattice represents downregulated genes. The colour scale presents the log 2-fold change value of DEGs. (JPG) [file pone.0275503.s005.jpg]

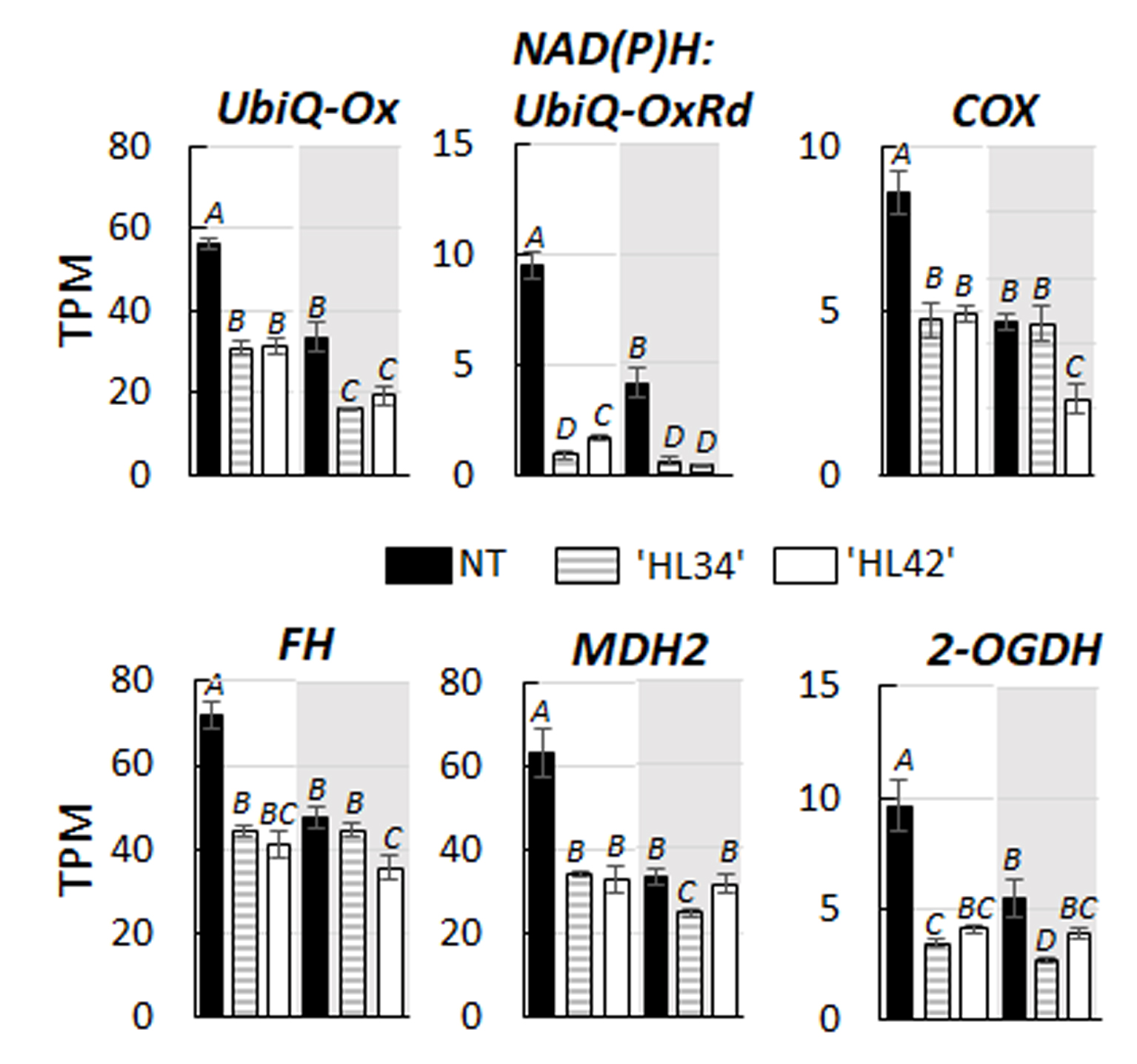

Supplement: S6 Fig — Plants included non-transformed (NT) control were grown under standard (white graph area) and low (shading graph area) irradiance levels. Data represent the means of normalized transcript per million (TPM) with the error bar of the SE. A-DAlphabets indicated significant differences (p < 0.05, n = 3). (JPG) [file pone.0275503.s006.jpg]

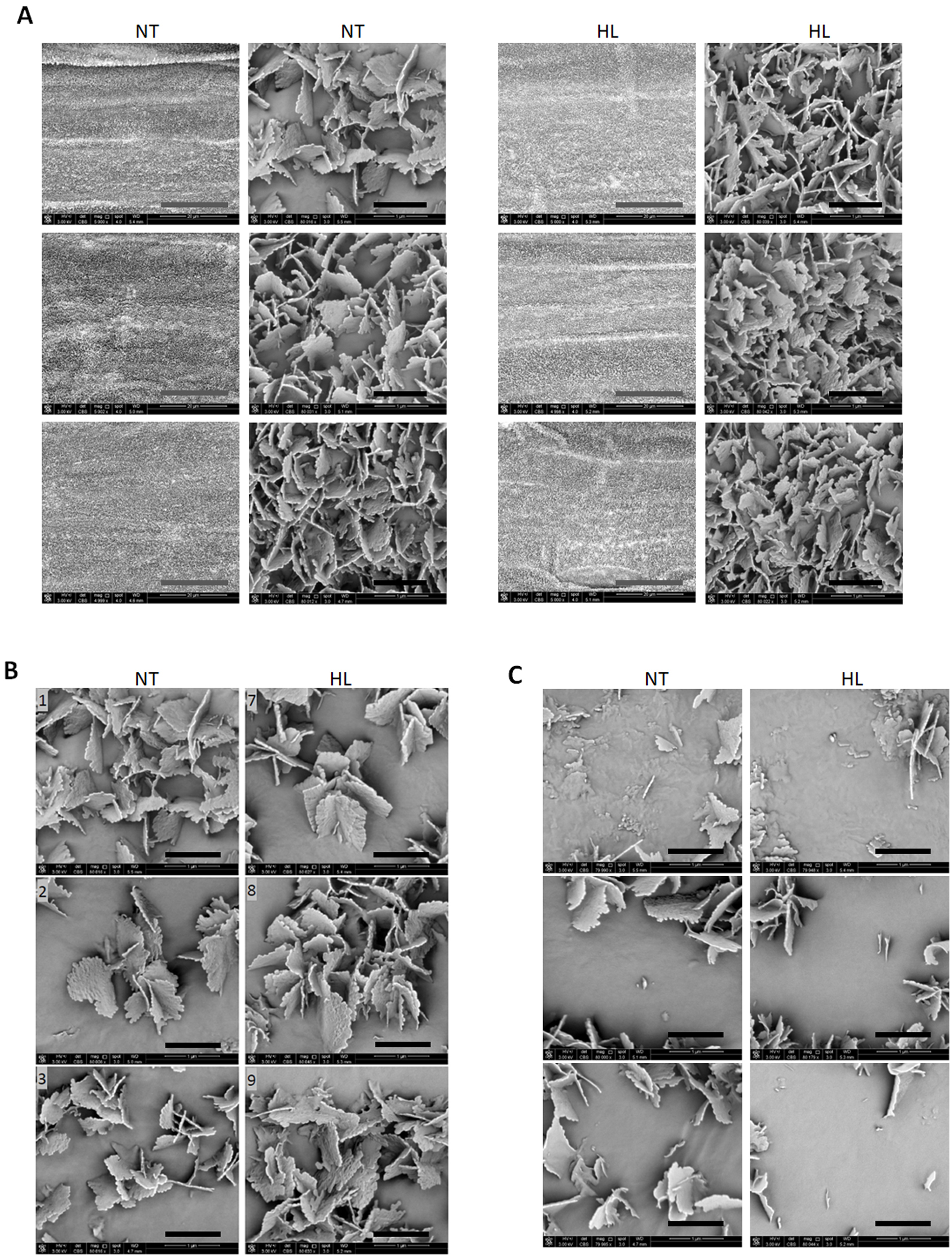

Supplement: S7 Fig — Wax crystal were compared between non-transformant control (NT) and high lipid (HL) Lolium. A Images were taken from the high dense area with two different magnifications. B Images from less dense areas. C Epicuticular wax film comparisons. (JPG) [file pone.0275503.s007.jpg]
