# Supplementary material for: Insight into the regulatory networks underlying the high lipid perennial ryegrass growth under different irradiances
Source: PLoS One. 2022 Oct 13;17(10):e0275503. doi: 10.1371/journal.pone.0275503 (PMC9560171; doi:10.1371/journal.pone.0275503)
Supplement: S1 Table — Locus identifier indicates the best-hit identification of the differentially expressed contigs from BLAST searches of the Oryza sativa IRGSP-1.0 databases. Lp-elF-4α and Lp-TEF1 were used as reference genes in this study. (DOCX) [file pone.0275503.s008.docx]

**S1 Table. List of primers and their respective sequences for quantitative RT-PCR of perennial ryegrass genes.**

Locus identifier indicates the best-hit identification of the differentially expressed contigs from BLAST searches of the *Oryza sativa* IRGSP-1.0 databases. *Lp-elF-4α* and *Lp-TEF1* were used as reference genes in this study.

| **Gene** | **Locus identifier** | **Primer** | **Primer sequence** |
| --- | --- | --- | --- |
| Eukaryotic initiation factor 4α | GO924770 | *Lp-eIF-4α-F* | CTCAACTTGAAGTGTTGGAGTG |
|  |  | *Lp-eIF-4α-R* | GATCTGGTCCTGGAAAGAATATG |
| Transcription elongation factor 1 | GR522099 | *Lp-TEF1-F* | CGTGTGATCGAGAGGTTTGA |
|  |  | *Lp-TEF1-R* | CGAATTTCCAGAGGGCAATA |
| Hexokinase 7 | XM_015782068 | *Lp-HK7-F* | CCAGGGTTTAGGCTCTCCTC |
|  |  | *Lp-HK7-R* | GGAAAAGGCAAGCGACATAC |
| SnRK1 subunit γ | XM_015760575 | *Lp-SnRK1-γ-F* | CGCCCATAAGAAATGTCGAT |
|  |  | *Lp-SnRK1-γ-R* | GATCCAACTGCAGAACCAG |
| Trehalose 6-phosphate synthase 1 | XM_015784904 | *Lp-TPS1-F* | CAACCCTTGTGAGCTTTCCC |
|  |  | *Lp-TPS1-R* | CTGCTTCGTTCTGTGCTCTG |
| Trehalose 6-phosphate synthase 6 | XM_015756424 | *Lp-TPS6-F* | GAAGCTCAGCAACCTTGACC |
|  |  | *Lp-TPS6-R* | GCCTCAAATACGAGTCGCAG |
| Trehalose 6-phosphate phosphatase 7 | XM_015795963 | *Lp-TPP7-F* | CGGTCGGTATACATCCCCAG |
|  |  | *Lp-TPP7-R* | CTCATCATCCGAACAAGGCG |
| Ubiquinol oxidase 1a, mitochondrial | XM_015779927 | *Lp-Ubiq-Ox-F* | CATCACAGTCCAGGGTGTCT |
|  |  | *Lp-Ubiq-Ox-R* | GTTGTCGATCTTGCCATCCT |
| NAD(P)H-ubiquinone oxidoreductase, mitochondrial | XM_015782427 | *Lp-Ubiq-OxRd-F* | GCCCTTGCTCAAGTTGACTC |
|  |  | *Lp-Ubiq-OxRd-R* | CTGCACCCCTGATCCTTAGA |
| Fumarate dehydrogenase 1, mitochondrial | XM_015777653 | *Lp-FH-F* | CGGCACCACTACTCTCAACA |
|  |  | *Lp-FH-R* | CACTGGCTTGAACACCAA |
| Malate dehydrogenase, mitochondrial | XM_015766118 | *Lp-MDH2-F* | GTACTGCCCCAACGCTCTTA |
|  |  | *Lp-MDH2-R* | CTGGCACGAACAACATCAAG |
| 2-Oxoglutarate dehydrogenase, mitochondrial | XM_015790994 | *Lp-2-OGDH-F* | GTACATCTTTGGCTGCGTGA |
|  |  | *Lp-2-OGDH-R* | GACGATCTGGAGGCTGTTGT |
| L-ascorbate oxidase | XM_015755566 | *Lp-AO-F* | CTTGGTCTTCGTTGGTGAGC |
|  |  | *Lp-AO-R* | CCCAGGCATGACGGTGAATA |
| L-ascorbate peroxidase 1, cytosolic | XM_015775012 | *Lp-APX-F* | CCGCAGCATATTTCTCCACG |
|  |  | *Lp-APX-R* | GACCCTGGACAAAGAACCCT |
