# Supplementary material for: Insight into the regulatory networks underlying the high lipid perennial ryegrass growth under different irradiances
Source: PLoS One. 2022 Oct 13;17(10):e0275503. doi: 10.1371/journal.pone.0275503 (PMC9560171; doi:10.1371/journal.pone.0275503)
Supplement: S2 Table — (DOCX) [file pone.0275503.s009.docx]

**S2 Table. Composition of wax compounds in high-lipid (HL) Lolium and non-transformant (NT) control.**

| *n = 10* | Content (mg kg^-1^ DW) | |  |  | Relative composition (%) | |  |  |
| --- | --- | --- | --- | --- | --- | --- | --- | --- |
| Compound | NT | HL | LSD | *p-Value* | NT | HL | LSD | *p-Value* |
|  |  |  |  |  |  |  |  |  |
| C20 fatty alcohol | 8.56 | 20.25 | 2.92 | *1.20E-07* | 0.12 | 0.23 | 0.03 | *9.79E-07* |
| C22 fatty alcohol | 15.25 | 19.57 | 2.82 | *0.0049* | 0.22 | 0.22 | 0.03 | *0.556* |
| C24 fatty alcohol | 36.66 | 86.13 | 10.80 | *5.55E-09* | 0.52 | 0.98 | 0.10 | *3.00E-08* |
| C25 fatty alcohol | 344.69 | 469.94 | 91.13 | *0.0098* | 4.77 | 5.34 | 0.32 | *0.0866* |
| C26 fatty alcohol | 3256.44 | 4078.34 | 410.38 | *0.0053* | 46.03 | 46.56 | 1.89 | *0.561* |
| C27 fatty alcohol | 540.82 | 614.87 | 83.24 | *0.078* | 7.60 | 7.02 | 0.47 | *0.0186* |
| C28 fatty alcohol | 877.34 | 943.06 | 149.57 | *0.368* | 12.44 | 10.78 | 1.49 | *0.0307* |
| C29 fatty alcohol | 73.18 | 69.34 | 12.10 | *0.514* | 1.05 | 0.79 | 0.16 | *0.0035* |
| C25 alkane | 35.99 | 34.43 | 3.78 | *0.512* | 0.50 | 0.38 | 0.07 | *0.0012* |
| C26 alkane | 13.81 | 9.30 | 3.17 | *0.0079* | 0.20 | 0.11 | 0.04 | *0.0001* |
| C27 alkane | 75.71 | 76.23 | 11.95 | *0.929* | 1.07 | 0.87 | 0.04 | *0.0002* |
| C28 alkane | 22.44 | 19.50 | 4.05 | *0.143* | 0.32 | 0.22 | 0.04 | *0.0002* |
| C29 alkane | 248.65 | 276.50 | 69.55 | *0.411* | 3.44 | 3.13 | 0.52 | *0.225* |
| C30 alkane | 23.94 | 25.51 | 4.49 | *0.472* | 0.34 | 0.29 | 0.03 | *0.0052* |
| C31 alkane | 305.60 | 397.70 | 64.99 | *0.0081* | 4.32 | 4.54 | 0.63 | *0.480* |
| C32 alkane | 10.91 | 11.68 | 2.49 | *0.522* | 0.15 | 0.13 | 0.02 | *0.105* |
| C33 alkane | 165.95 | 207.98 | 12.97 | *0.0046* | 2.34 | 2.37 | 0.20 | *0.728* |
| Aldehydes | 363.67 | 411.63 | 116.08 | *0.399* | 5.01 | 4.67 | 1.03 | *0.497* |
| Fatty acids | 117.37 | 159.71 | 44.39 | *0.0604* | 1.68 | 1.81 | 0.48 | *0.574* |
| Alkyl esters | 110.86 | 100.79 | 34.29 | *0.545* | 1.55 | 1.15 | 0.35 | *0.0256* |
| Triterpenoid-amyrin | 233.02 | 521.90 | 44.91 | *7.30E-11* | 3.30 | 5.98 | 0.52 | *2.88E-09* |
| Epicuticular wax (% DW) | 0.71 | 0.88 | 0.10 | *0.0017* |  |  |  |  |
