# Supplementary material for: Insight into the regulatory networks underlying the high lipid perennial ryegrass growth under different irradiances
Source: PLoS One. 2022 Oct 13;17(10):e0275503. doi: 10.1371/journal.pone.0275503 (PMC9560171; doi:10.1371/journal.pone.0275503)
Supplement: S1 Protocol — The diurnal experiment was conducted with ten clonal plantlets each of five segregation progenies of T2 homozygous HL Lolium and five segregated null plants (cultivar ’Alto’ x ’Elite 50’, PGG Wrightson, NZ). Homozygous HL Lolium with ‘Alto’ background were recently generated through a commercial breeding programme and used for field trials in the Midwest of the United States of America [30]. Therefore, this supplementary experiment was conducted using the ‘Alto’ lines as ‘preferred field-tested material’ than the previous generated HL Lolium with ‘Impact’ background (laboratory-tested material). After the last synchronization round, plants were grown through an ’establishment period’ in washed coarse sand (as described in the main experiments) in a controlled environment room with standard light. At the end of this ’establishment period’, all plantlets were defoliated to 5–6 cm above the sand surface and regrown for an additional three weeks with similar treatment as described in the main experiments under standard light. Plants were destructively harvested at the start of daylight (0 h) through the next day (24 h) with 4 h time-point intervals (S1A Fig) and divided into 5 organs (S1B Fig): Leaf tip, leaf middle, ligule, sheath and root (cleaned). Plant materials were freeze-dried for 3–4 d and stored at -80 °C until use for sugars and FA analysis (S1C and S1D Fig). (PDF) [file pone.0275503.s012.pdf]

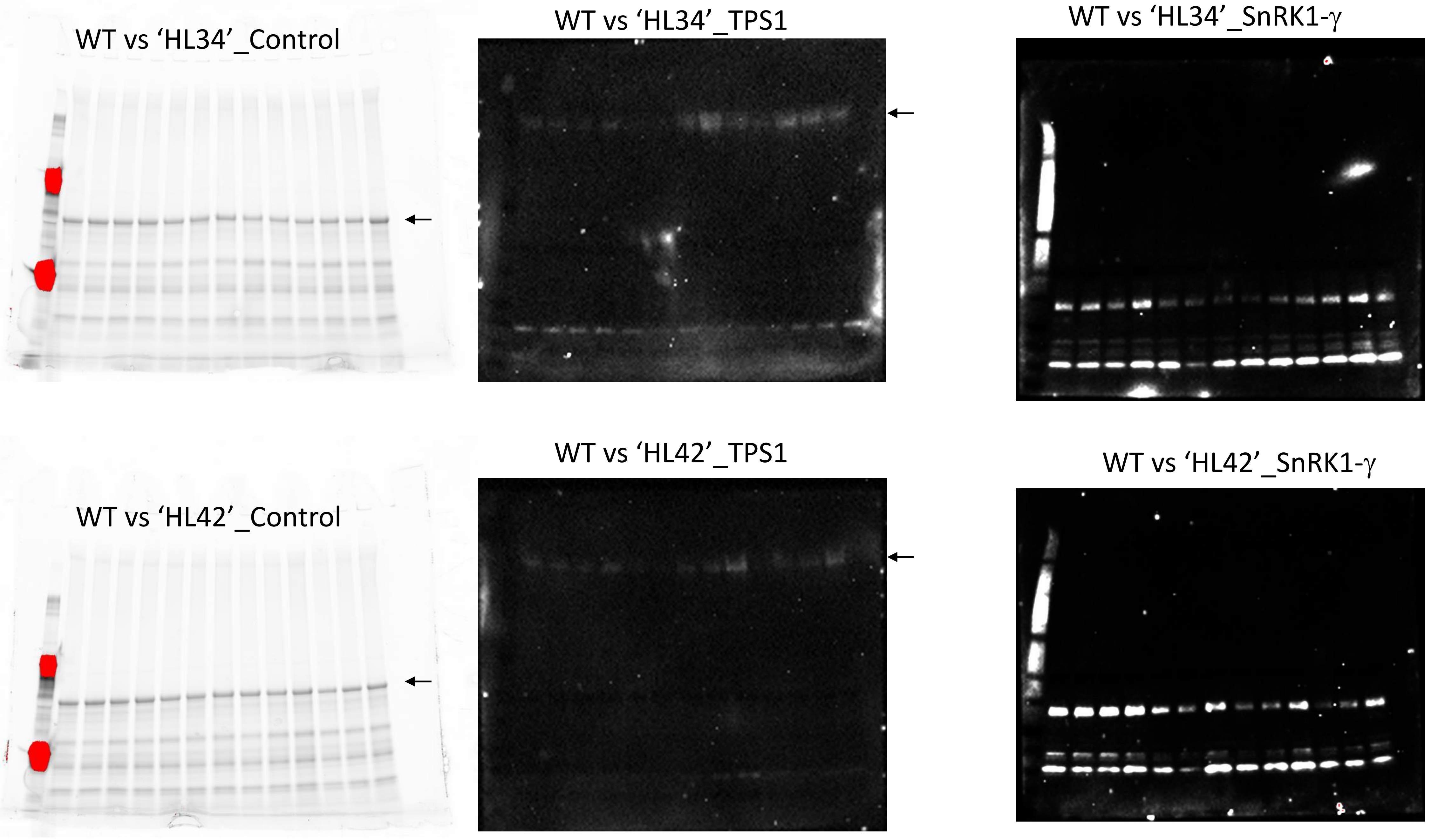
WT vs ‘HL34’_Control
WT vs ‘HL34’_TPS1
WT vs ‘HL34’_SnRK1-γ
WT vs ‘HL42’_Control
WT vs ‘HL42’_TPS1
WT vs ‘HL42’_SnRK1-γ

Fig 4

WT vs ‘HL34’_APX

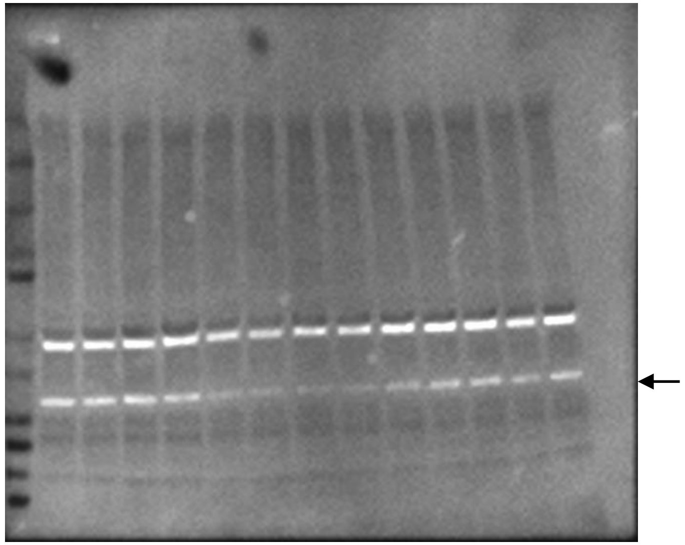

WT vs ‘HL34’_AO

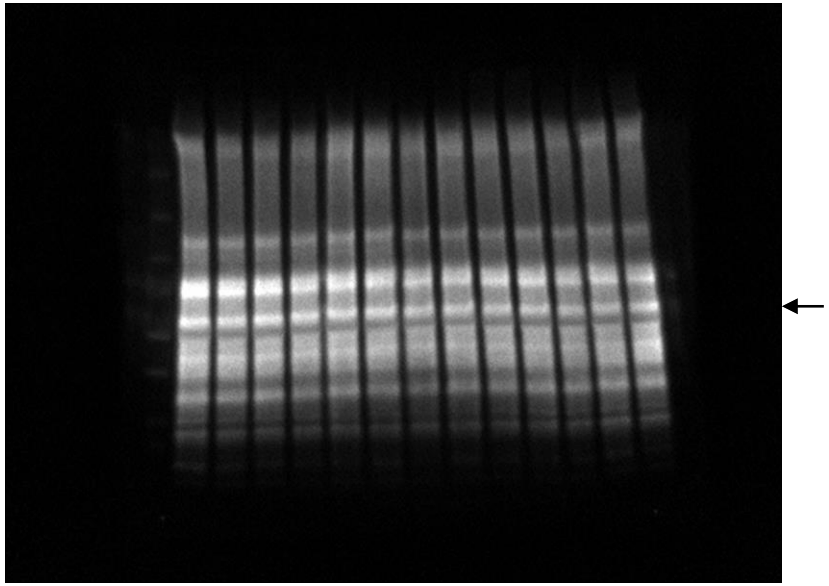

WT vs ‘HL42’_APX

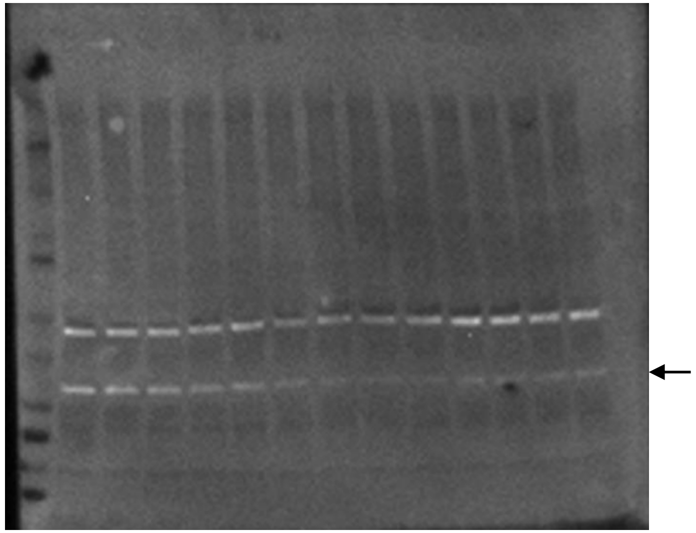

WT vs ‘HL42’_AO

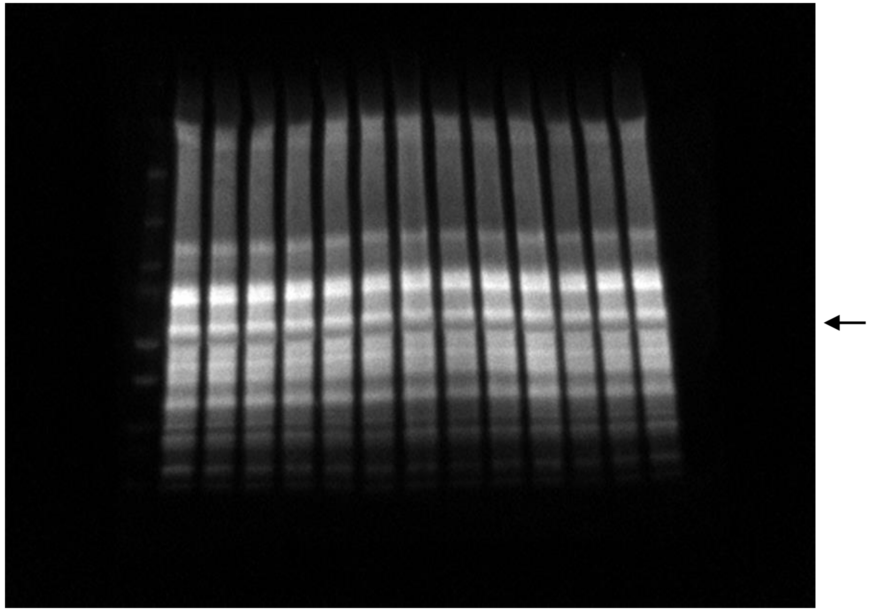

Fig 4

WT vs ‘HL34’_MDH2

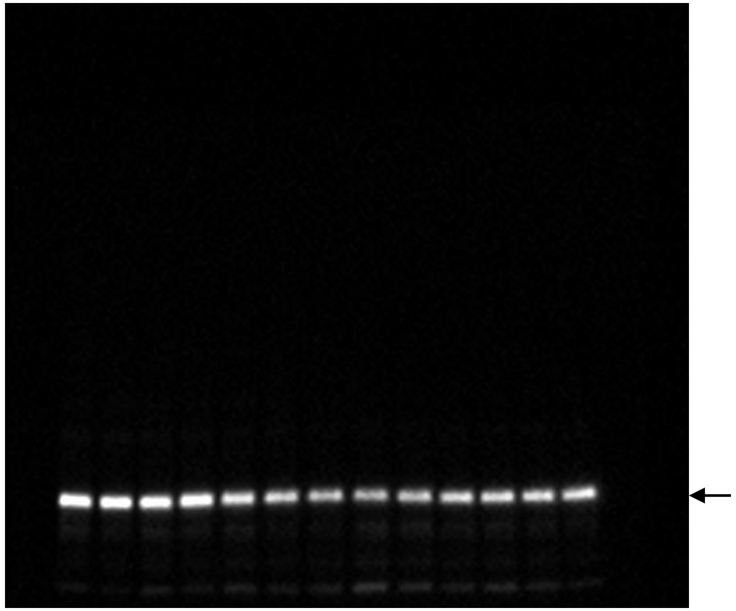

WT vs ‘HL34’_COX

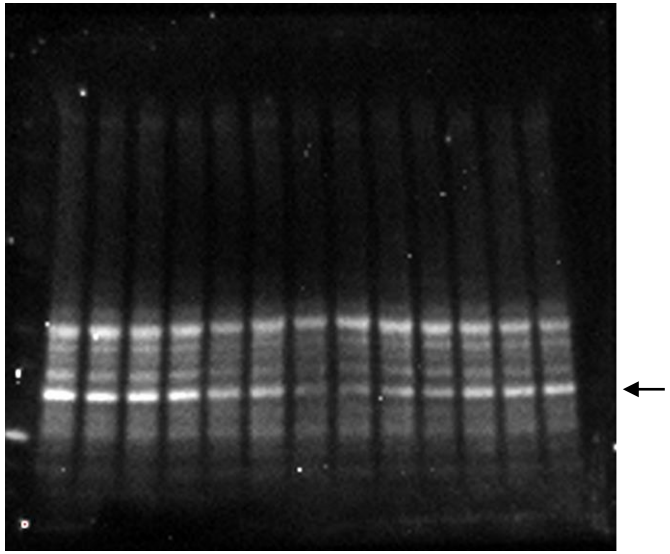

WT vs ‘HL42’_MDH2

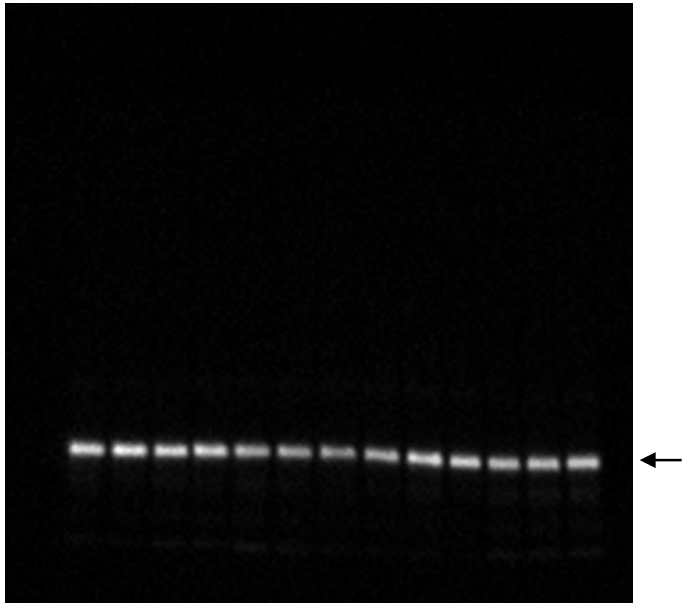

WT vs ‘HL42’_COX

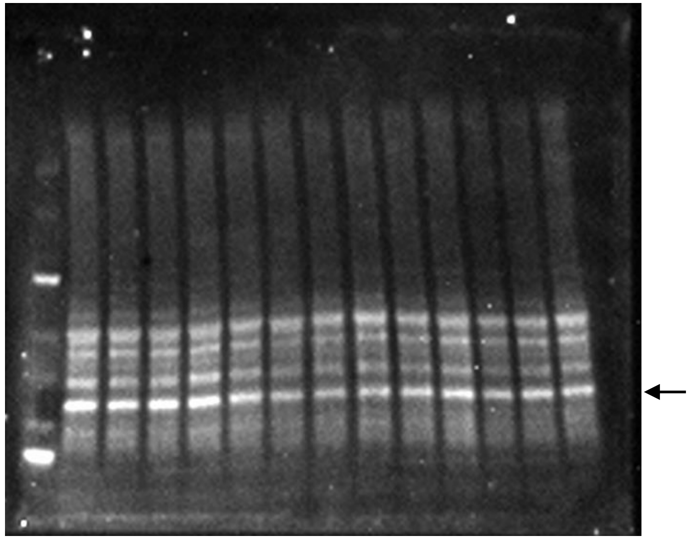

Fig 4

“HL30’_stain free gel LOW

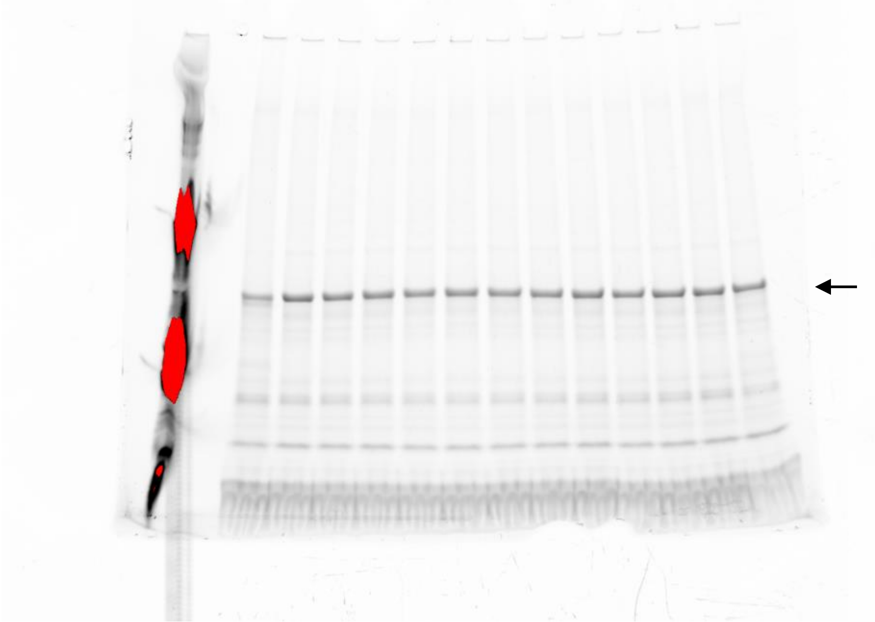

“HL34’_stain free gel LOW

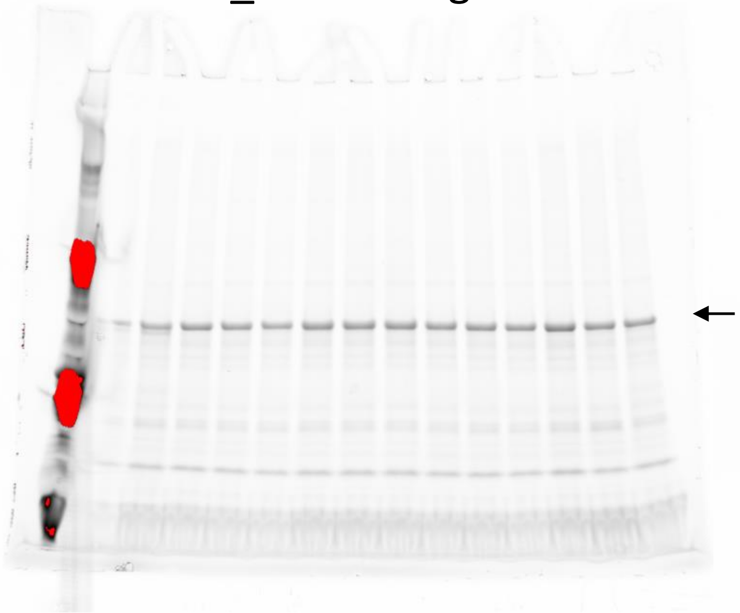

“HL42’_stain free gel LOW

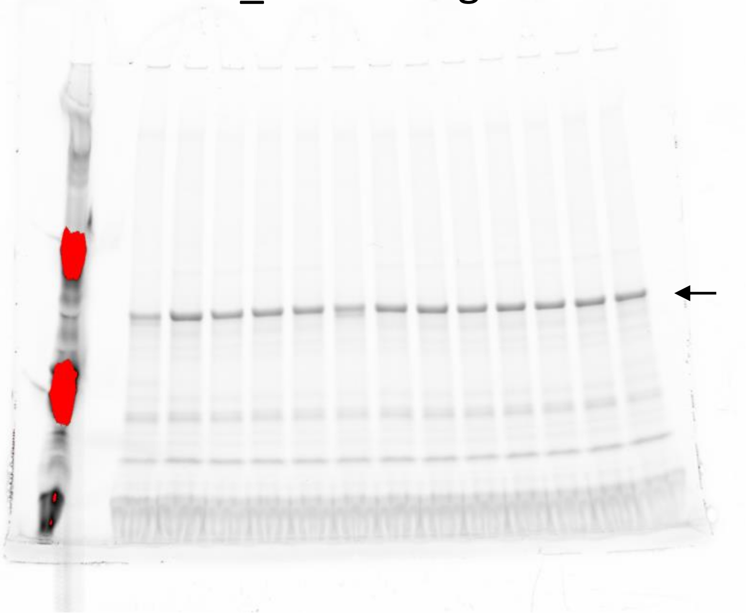

“HL30’_stain free gel STD

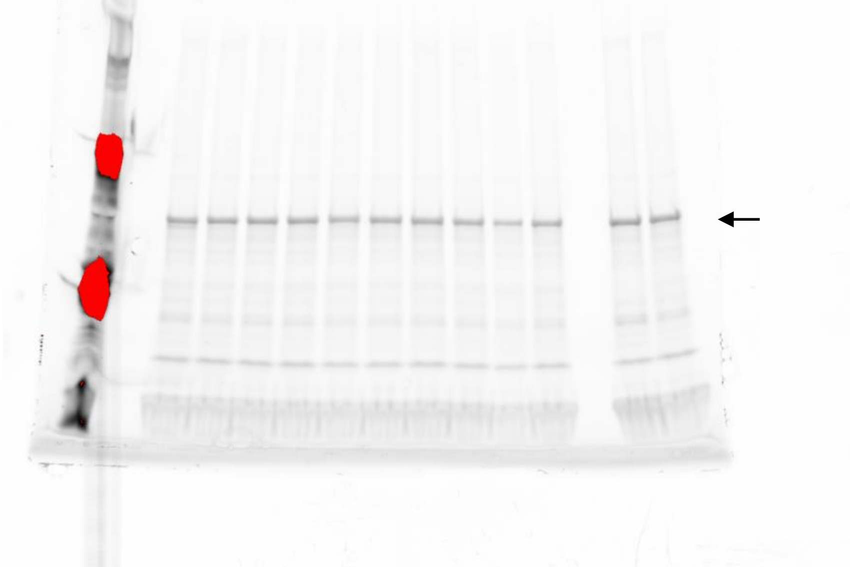

“HL34’_stain free gel STD

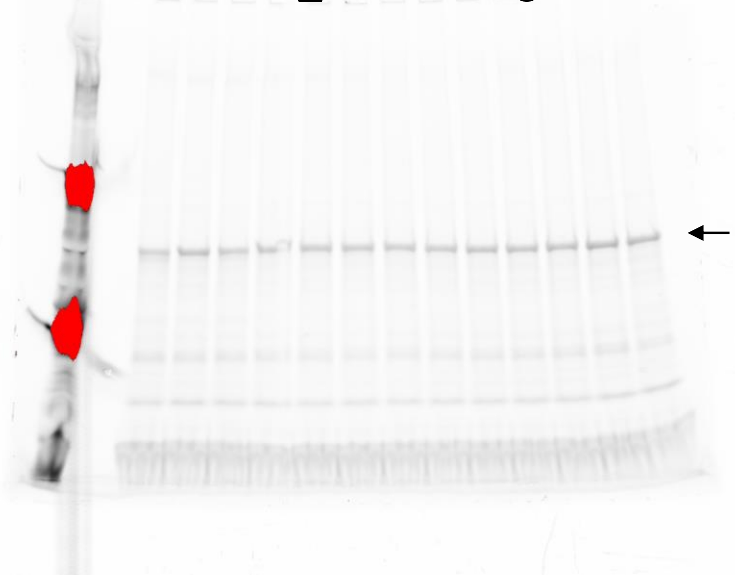

“HL42’_stain free gel STD

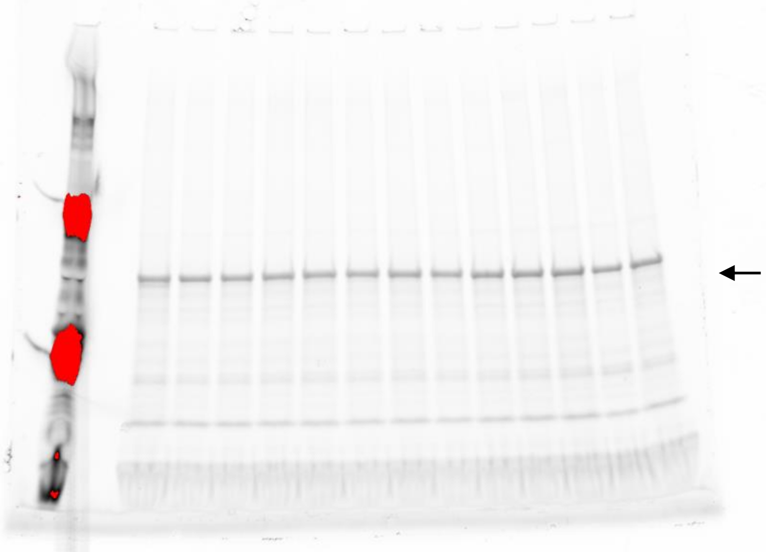

Fig S2

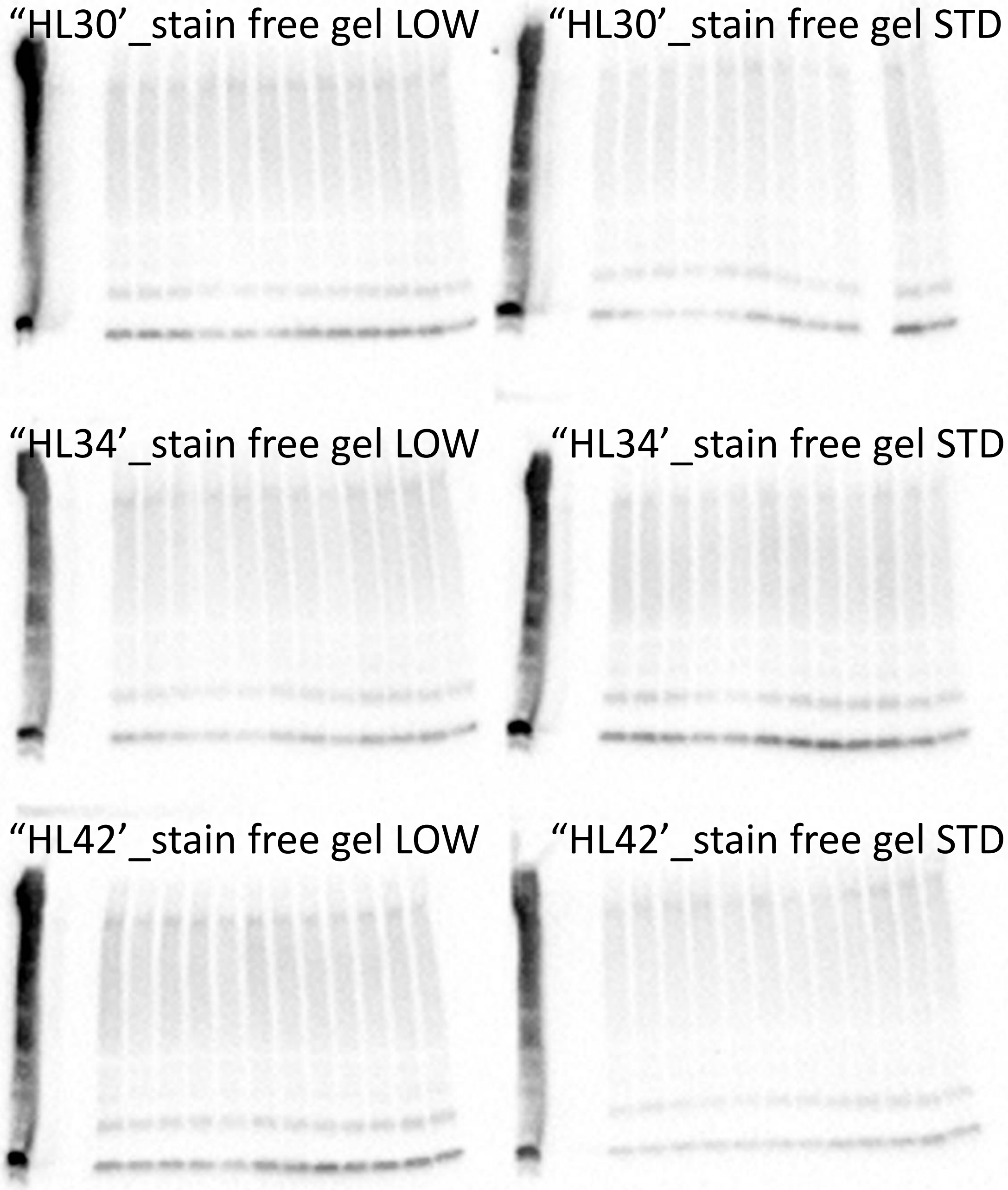
“HL30’_stain free gel LOW
“HL30’_stain free gel STD
“HL34’_stain free gel LOW
“HL34’_stain free gel STD
“HL42’_stain free gel LOW
“HL42’_stain free gel STD

Fig S2

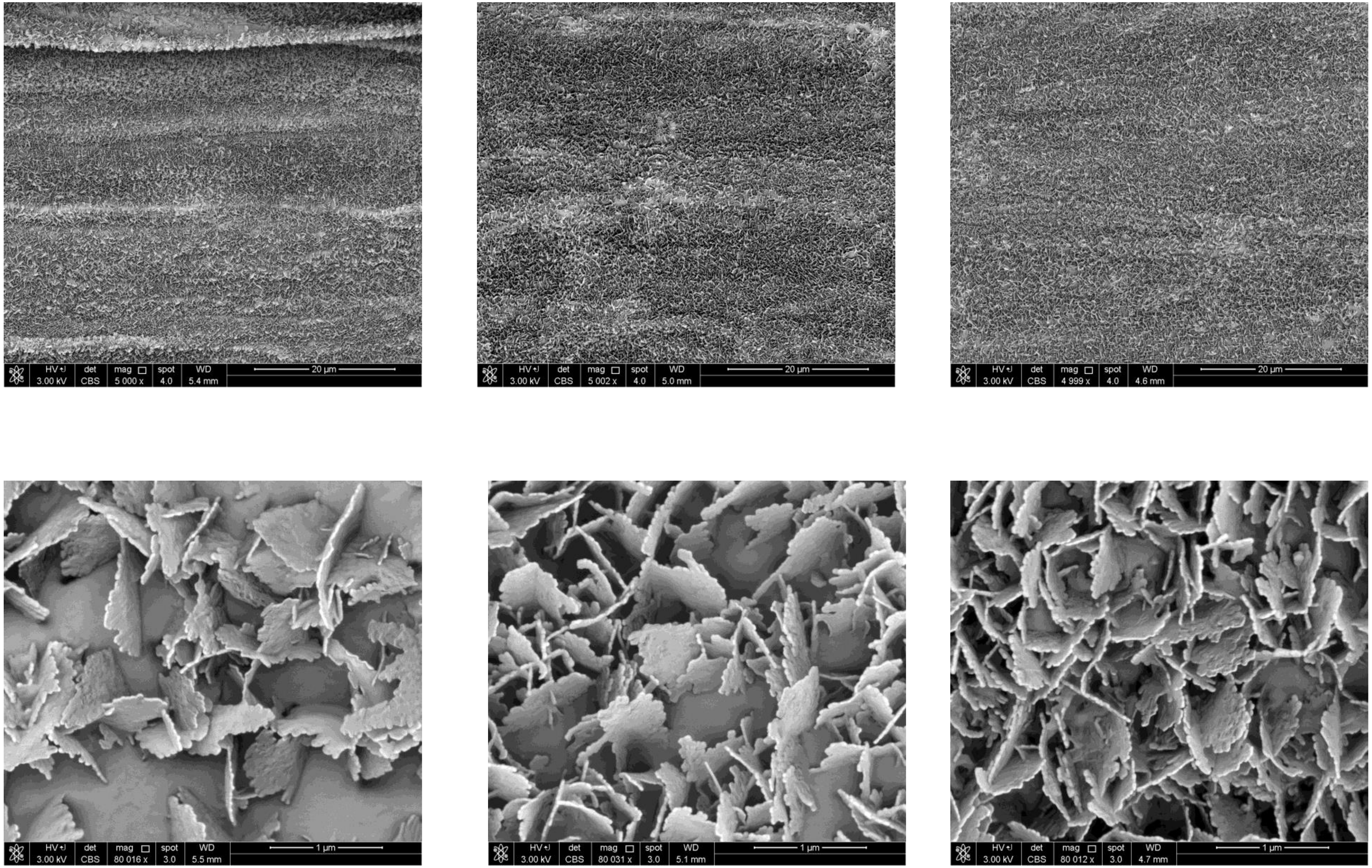
HV 3.00 kV det CBS mag 5 000 x spot 4.0 WD 5.4 mm 20 µm
HV 3.00 kV det CBS mag 5 002 x spot 4.0 WD 5.0 mm 20 µm
HV 3.00 kV det CBS mag 4 999 x spot 4.0 WD 4.6 mm 20 µm
HV 3.00 kV det CBS mag 80 016 x spot 3.0 WD 5.5 mm 1 µm
HV 3.00 kV det CBS mag 80 031 x spot 3.0 WD 5.1 mm 1 µm
HV 3.00 kV det CBS mag 80 012 x spot 3.0 WD 4.7 mm 1 µm

NT

Fig S7A

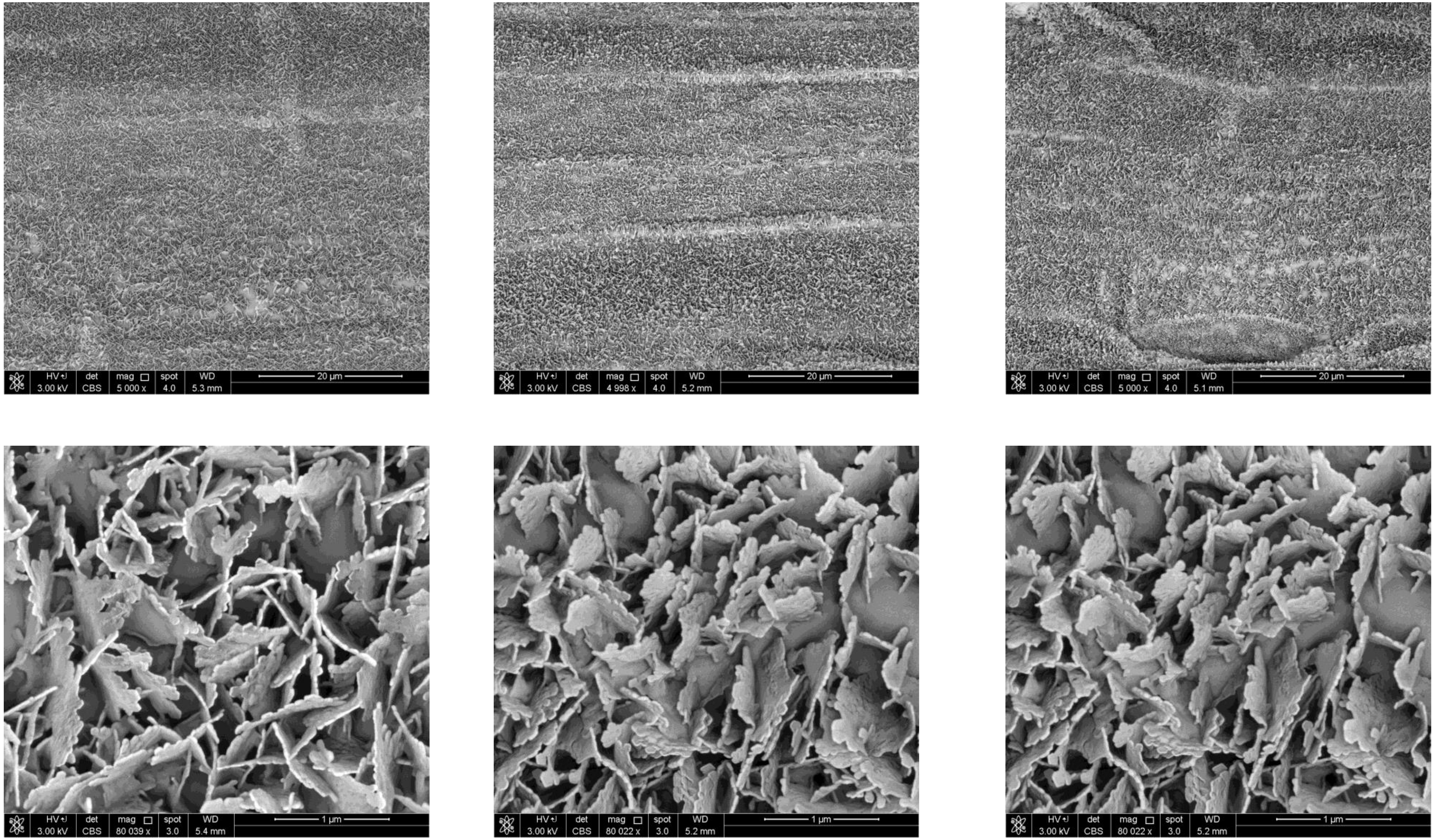
HV 3.00 kV det CBS mag 5 000 x spot 4.0 WD 5.3 mm
20 µm
HV 3.00 kV det CBS mag 4 998 x spot 4.0 WD 5.2 mm
20 µm
HV 3.00 kV det CBS mag 5 000 x spot 4.0 WD 5.1 mm
20 µm
HV 3.00 kV det CBS mag 80 039 x spot 3.0 WD 5.4 mm
1 µm
HV 3.00 kV det CBS mag 80 022 x spot 3.0 WD 5.2 mm
1 µm
HV 3.00 kV det CBS mag 80 022 x spot 3.0 WD 5.2 mm
1 µm

HL

Fig S7A

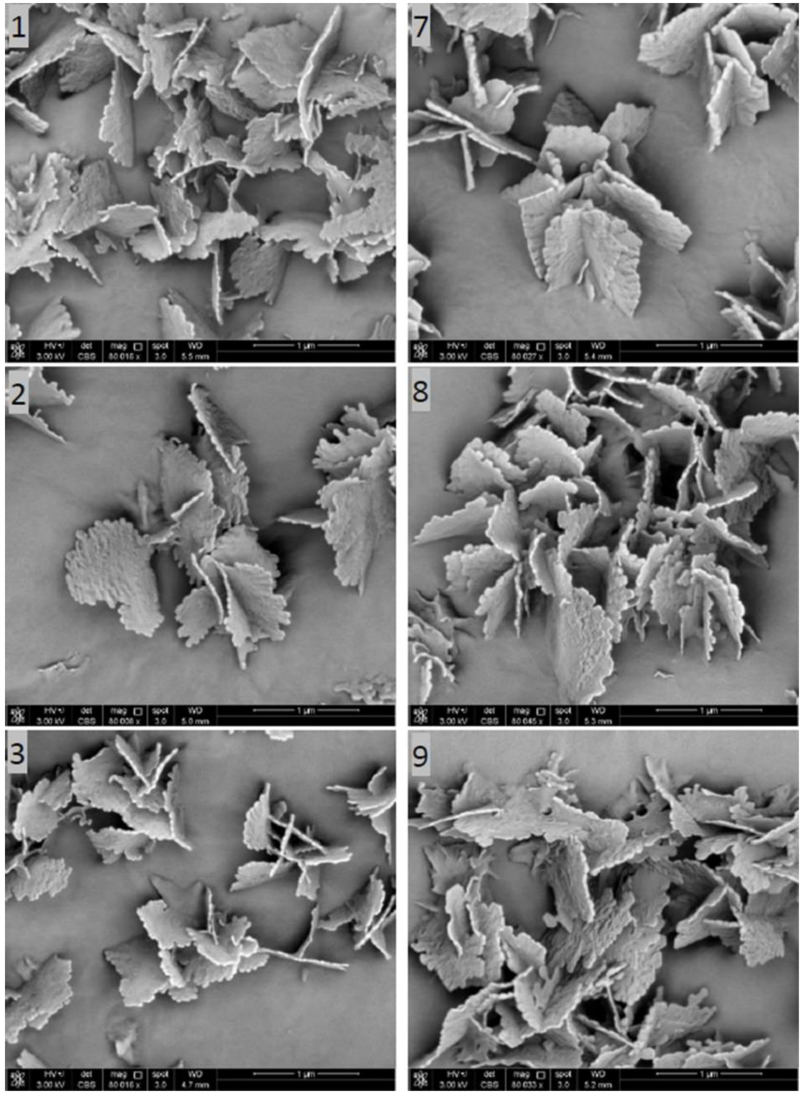

Fig S7B

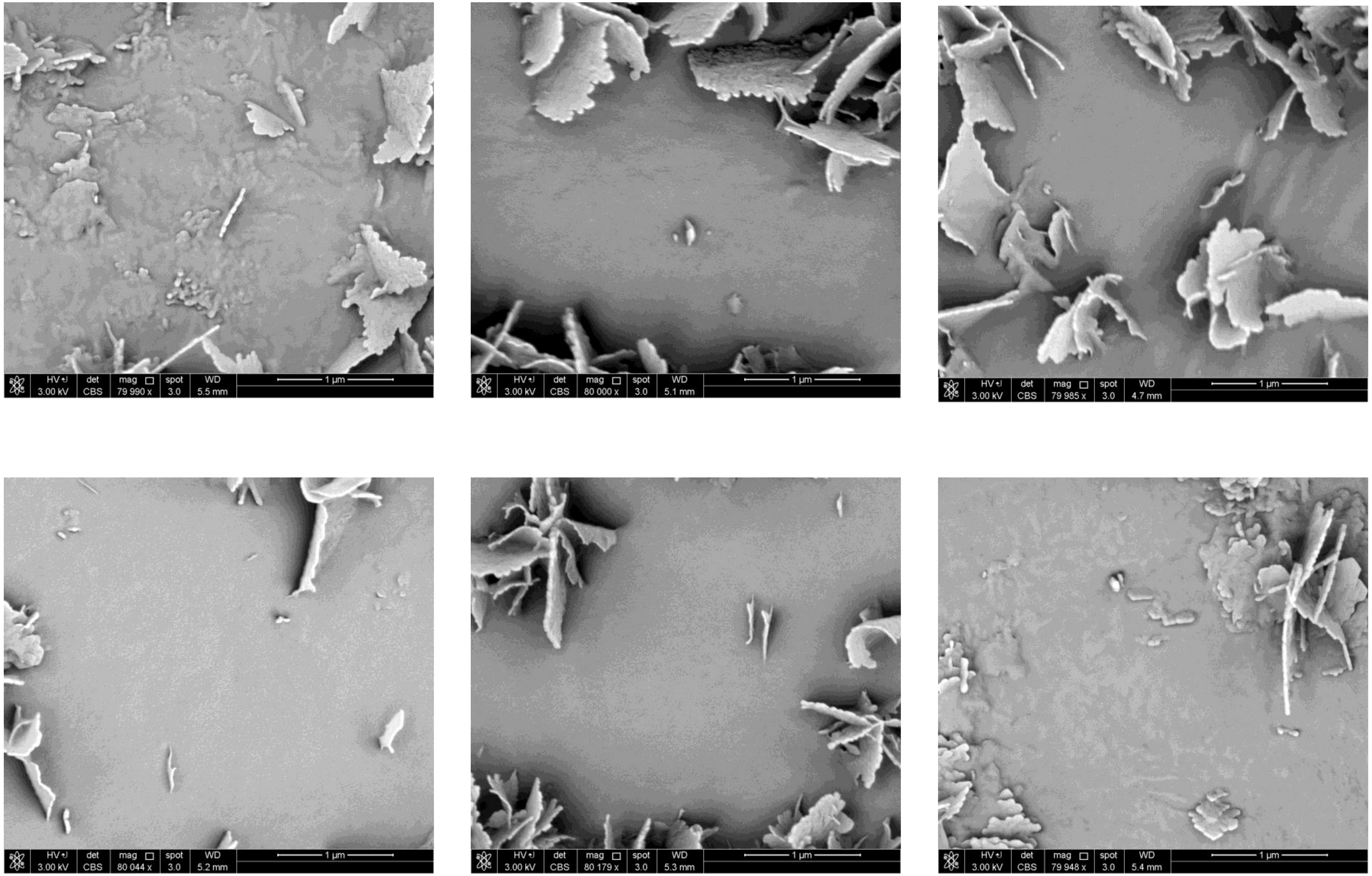
HV 3.00 kV det CBS mag 79 990 x spot 3.0 WD 5.5 mm 1 µm
HV 3.00 kV det CBS mag 80 000 x spot 3.0 WD 5.1 mm 1 µm
HV 3.00 kV det CBS mag 79 985 x spot 3.0 WD 4.7 mm 1 µm
HV 3.00 kV det CBS mag 80 044 x spot 3.0 WD 5.2 mm 1 µm
HV 3.00 kV det CBS mag 80 179 x spot 3.0 WD 5.3 mm 1 µm
HV 3.00 kV det CBS mag 79 948 x spot 3.0 WD 5.4 mm 1 µm

NT

HL

Fig S7C
